# Variability of heat stress using the UrbClim climate model in the city of Seville (Spain): mitigation proposal

**DOI:** 10.1007/s10661-023-11768-8

**Published:** 2023-09-07

**Authors:** David Hidalgo-García, Hamed Rezapouraghdam

**Affiliations:** 1 Technical Superior School of Building Engineering, University of Granada, Fuentenueva Campus, 18071 Granada, Spain; 2 Faculty of Tourism, Eastern Mediterranean University, Via Mersin 10 Turkiye, 99628 Gazimagusa, TRNC Turkey

**Keywords:** Heat mitigation, Heat stress index, Heat wave, UrbClim model

## Abstract

Climate change is creating an increase in temperatures, which is harming the quality of life of people all over the world, particularly those with minimal financial resources. While 30% of the world’s population is now vulnerable to extreme heat, estimates show that ratio will rise to 74% in the next 20 years, according to forecasts. Using the UrbClim climate model, this study examines the space-time variability of the heat stress index (HI) in different local climate zones (LCZs), as well as how heat wave conditions might affect this index based on land use and land cover. To that end, Seville, in Southern Spain, was investigated during the summer of 2017, when it had four heat waves. The following indices were considered for each urban sub-area: Normalized Difference Vegetation, Proportion Vegetation, Normalized Difference Built, and Urban Index. The goal is to conduct a statistical analysis of the link between the aforementioned elements and the heat stress index in order to recommend mitigation and resilience techniques. Our findings showed that compact and industrial LCZs (2, 3, and 10) are less resistant to HI than open and rural regions (5, 6, B, D, and G), which are more resistant to HI due to higher vegetation rates. The heat wave condition exacerbates the HI in all LCZs. As a result, initiatives such as enhancing open space, increasing green space, or using green roofs and façades might alleviate heat stress and improve people’s quality of life.

## Introduction

The impact of extreme weather events connected to global warming is one of the most important concerns that humans are now confronting (Kovats et al., 2005; Song et al., 2020). The 6th Assessment Report of the Intergovernmental Panel on Climate Change (IPCC) has highlighted a significant increase in environmental temperatures in recent decades, which will have significant negative effects on people’s health and quality of life, particularly those who live in cities (IPCC, 2021). The alteration and modification of the soil as cities expand, in combination with population growth, cause an increase in global temperatures and contribute to climate change (Li et al., 2011). Changes on the earth’s surface involving impermeable materials reduce evapotranspiration (Stewart & Oke, 2012). These spaces absorb solar radiation and heat up during the day, and release the heat into the atmosphere at night (An et al., 2020; Arnfield, 2003; Zhou et al., 2015). The latest estimates of the United Nations Organization (UN) foresee a 20% increase in the urban population by the year 2050 (UN, 2018), which implies a significant transformation of urban coverage worldwide (Schneider et al., 2010). In turn, the phenomenon known as urban heat island (UHI) influences the rise in city temperatures. Extreme weather conditions such as heat waves can result from the intensity of such events, which can be influenced by human action. Numerous studies confirm that heat waves are becoming more intense and more frequent, and last longer (Coumou et al., 2013; Lau & Nath, 2012; Meehl & Tebaldi, 2004).

It is estimated that 30% of the world’s population currently suffers from extreme heat, and that figure may rise to 74% in the next 20 years (Mora et al., 2017). Given this climatic emergency, it is critical to conduct studies to determine which city areas are most vulnerable to severe heat stress. Urban areas with high temperatures are identified as urban hot spots (UHS). UHS have positive associations with various local climatic zones (LCZs) and land use/land cover (LULC) (Hidalgo & Arco, 2022; Amindin et al., 2021; Hidalgo & Arco, 2021). The scientific community has approved a number of metrics and calculation systems for measuring heat exposure in urban areas. For instance, wet bulb globe temperature, effective temperature, universal thermal climatic index (UTCI), humidex (HU), and heat index (HI) are among these metrics. HI, on the other hand, is one of the most widely used methods within the scientific community (Jacobs et al., 2019; Kotharkar et al., 2021; Verdonck et al., 2018) because it produces adequate results with only two parameters: ambient temperature and relative air humidity. One possibility for determining these environmental variables is to distribute temperature and humidity probes throughout the urban areas of study (Kotharkar et al., 2021; Kumar et al., 2022) or using urban climate models such as Muklimo from Germany’s meteorological agency (Geletič et al., 2018) or UrbClim from the European Space Agency’s Copernicus climate change service (De Ridder et al., 2015; Martí Ezpeleta & Royé, 2021; Verdonck et al., 2018). Since the last model provides climatic variables at a resolution of 100 m, it is widely used in studies of heat stress in urban areas (Royé et al., 2021; Verdonck et al., 2018).

Heat stress in cities has a high space-time variability and is influenced by climatic and morphological factors. A study of heat stress in Madrid using the UrbClim model between 2008 and 2017 found a significant positive correlation between the various LULCs and heat stress (Royé et al., 2021). The five Indian cities Kolkata, Chennai, Delhi, Mumbai (Kumar et al., 2022), and Nagpur (Kotharkar et al., 2021) reported increased heat stress in the face of an environmental heat wave situation. The increase is greater in areas with higher construction and population density than in neighborhoods with lower density and population. The classification of LCZs is commonly used in studies accounting for the morphological conditions of cities (Hidalgo & Arco, 2021; Ngarambe et al., 2020; Stewart & Oke, 2009; Wang & Ouyang, 2017). Accordingly, heat stress studies on the cities of Nagpur (India) (Kotharkar et al., 2021), Brno (Czech Republic) (Geletič et al., 2018), and Antwerp, Brussels, and Ghent (Belgium) (Verdonck et al., 2018) found that the LCZs identified as 2, 3, 5, 8, 9, and 10 presented greater heat stress, whereas LCZs 6, B, D, and G indicate less heat stress owing to more green spaces and smaller impervious areas. These studies, which are based on the average temperatures during a certain time period, are appropriate for understanding the global effects of heat on the population; however, they do not provide complete information on the periods when cities exceed average temperatures due to extreme weather events, such as heat waves.

The objective of this research is to determine the space-time variability of heat stress in the different LCZs and LULCs of Seville (Spain) during the summer of 2017, as well as how heat wave conditions may increase this value. To do so, the UrbClim model’s climatic variables of ambient temperature and relative humidity were used, and Sentinel 2 images were utilized to produce the Vegetation Proportion (PV), Normalized Difference Vegetation Index (NDVI), Built Normalized Difference Index (NDBI), and Urban Index (UI) for the various LCZs to determine their relationship with heat index variability. The evolution of the UHS in various LULCs and LCZs was then investigated. Finally, statistical analyses using the data panel and ANOVA techniques were used to measure correlations between the obtained data.

In this study, the following are our research questions: (1) What spatio-temporal variability does the heat stress index and UHS present in the different LCZs and LULCs of Seville? (2) How does this index intensify during environmental heat waves? (3) Is there a link between the heat stress index and the NDVI, PV, NDBI, and UI indices in the various LCZs? Our goal is to provide a panoramic view of the spatio-temporal variability of the heat stress index on the city of Seville’s various LCZs and LULCs, as well as to determine how heat wave conditions can increase this index. Extrapolation of open-access methodology results used in this research could aid decision-making by urban planners and public administrations for the development of new urban areas. The promotion of heat-resistant LCZs will assist urban areas in becoming climate change–resistant environments, thereby improving people’s quality of life.

## Materials and methods

### Study area

**Fig. 1:**
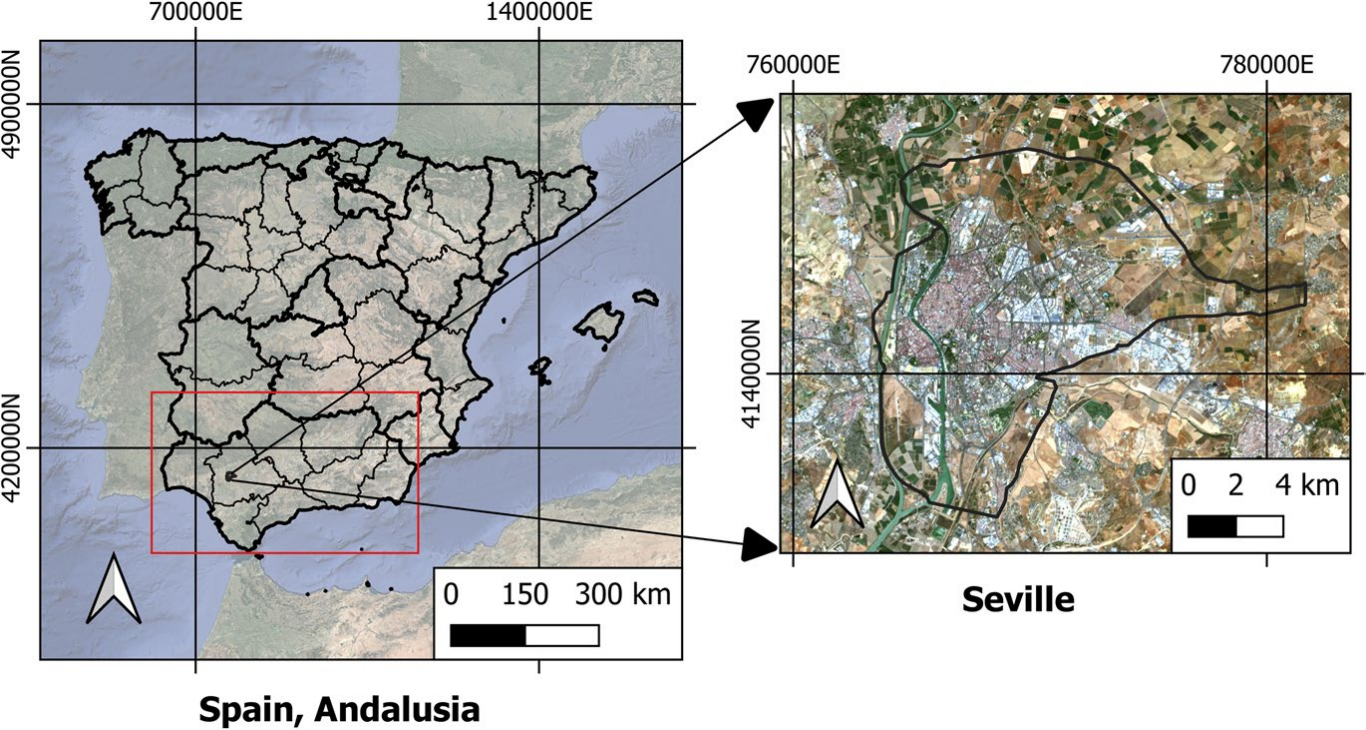
Study area, Spain, Andalusia, Seville

Seville (Fig. 1) is located in the Andalusia region of southern Spain. Seville has a 140-km^2^ urban area and a population of 688,711. However, the metropolitan area of Seville has a population of 1,548,741 inhabitants, making it the fourth most populous city in Spain. The UTM coordinates are *X*: 768772.582 and *Y*: 4137366.748; the city is located only 7 m above sea level. It has cold and humid winters and dry, warm summers according to the Köppen-Geiger Mediterranean climate typology (Csa). The proximity of the Mediterranean Sea, on the other hand, has a considerable influence on Seville’s climate. Throughout the year, temperatures oscillate between 3 and 36 degrees Celsius (°C), with highs of 40 °C or more in the summer. The city’s location allows for a very high number of annual hours of sunshine (3526), giving an average of 9.66 h per day.

### Methodology

**Fig. 2:**
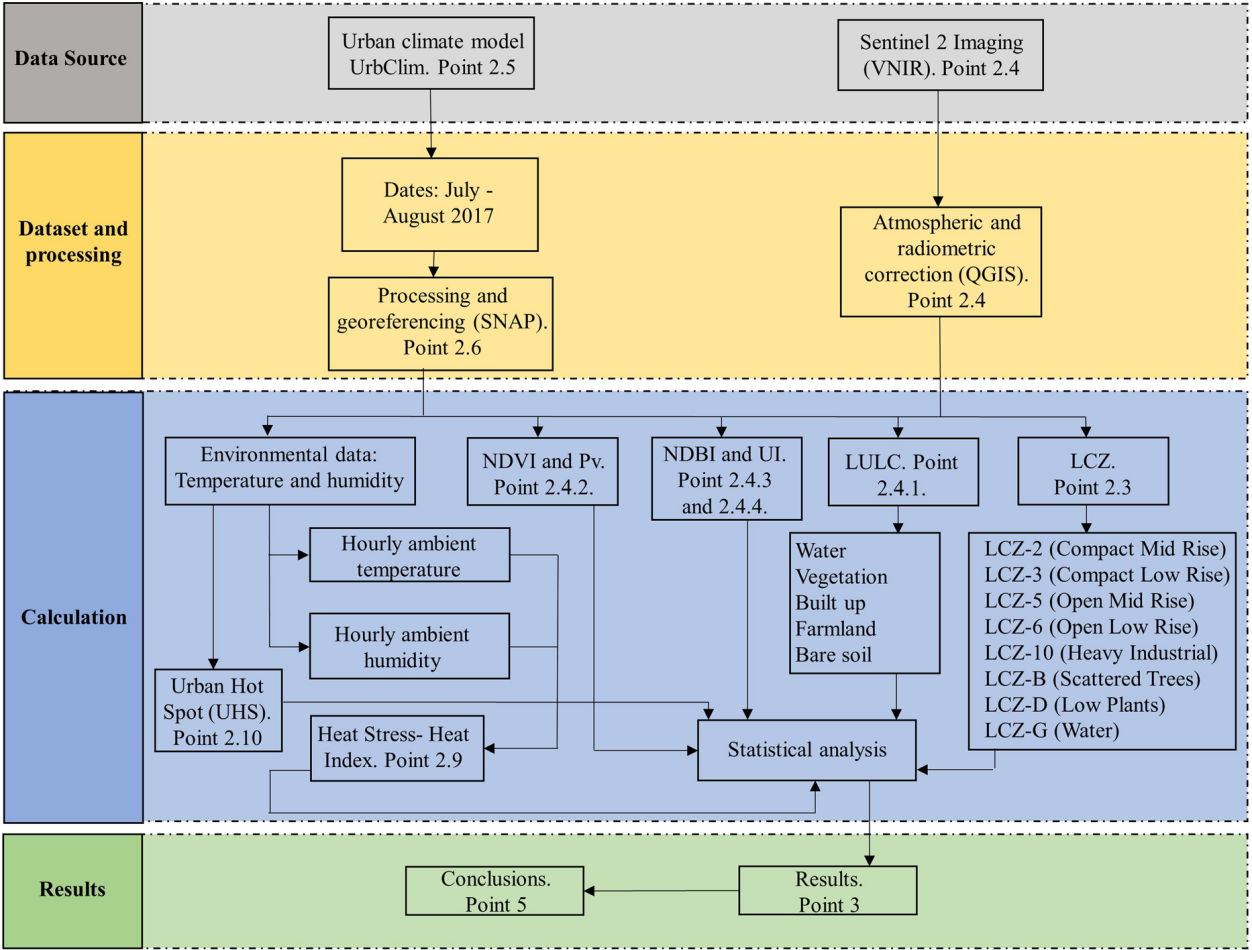
Methodology

Figure 2 depicts the methodology used in this study.

The PV, NDVI, UI, and NDBI indices of the city were determined at a resolution of 10 m using Sentinel 2 images. The first two indices are related to the amount of vegetation in an area and its conservation status. The last two are construction-related and enable territorial analysis in urban studies. Following that, the LULC maps were created. The support vector machine (SVM) methodology was used with QGIS software to classify the land cover, and the terrestrial cover was determined using a confusion matrix (Campbell, 1996). The application of this methodology in investigations requiring land surface classification is well documented in the literature (Yoo et al., 2019). Then, in order to characterize the landscape and urban structure, we identified the various LCZs that comprised the city. Using UrbClim data from the Copernicus Data Store (https://cds.climate.copernicus.eu/cdsapp#!/dataset/sis-urban-climate-cities?tab=form), the average values of ambient temperature and relative humidity in each LCZ were obtained for periods of heat wave and normal environmental conditions between July and August 2017. This year was selected because it is the last year for which UrbClim data was in the Copernicus Data Store. The heat stress index and the UHS in each LULC and LCZ were calculated using these values and then correlated with the other indices using statistical analysis software STATA, version 16.

### LCZ mapping and classification

**Fig. 3:**
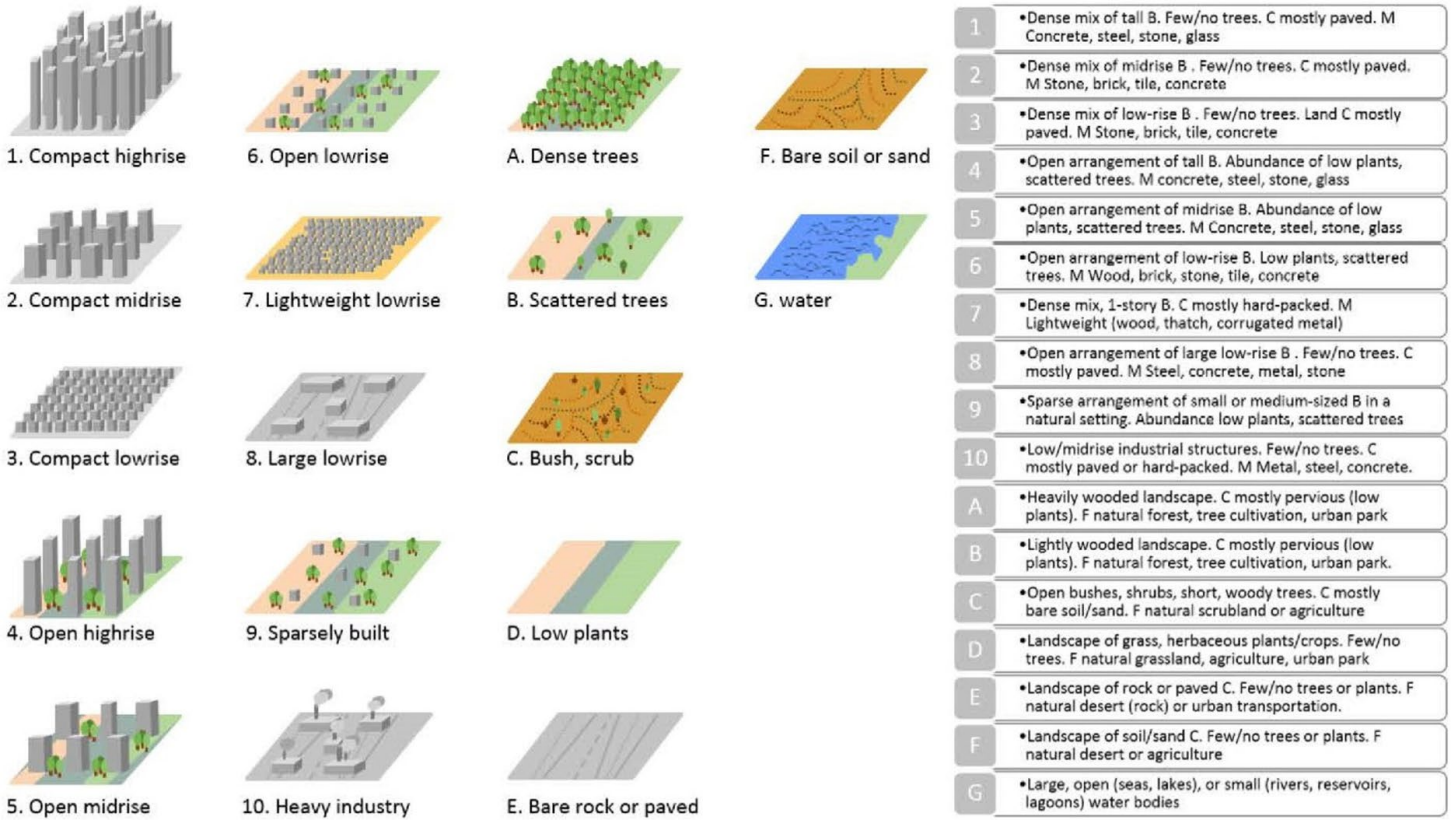
LCZ established by authors Stewart and Oke (2012)

In accordance with Stewart and Oke’s (2009) classification, we chose to divide the city of Seville into different LCZ spaces of similar coverage, morphology, and development (Fig. 3). This option was chosen over using pre-existing LCZ maps in order to achieve high precision.

**Fig. 4:**
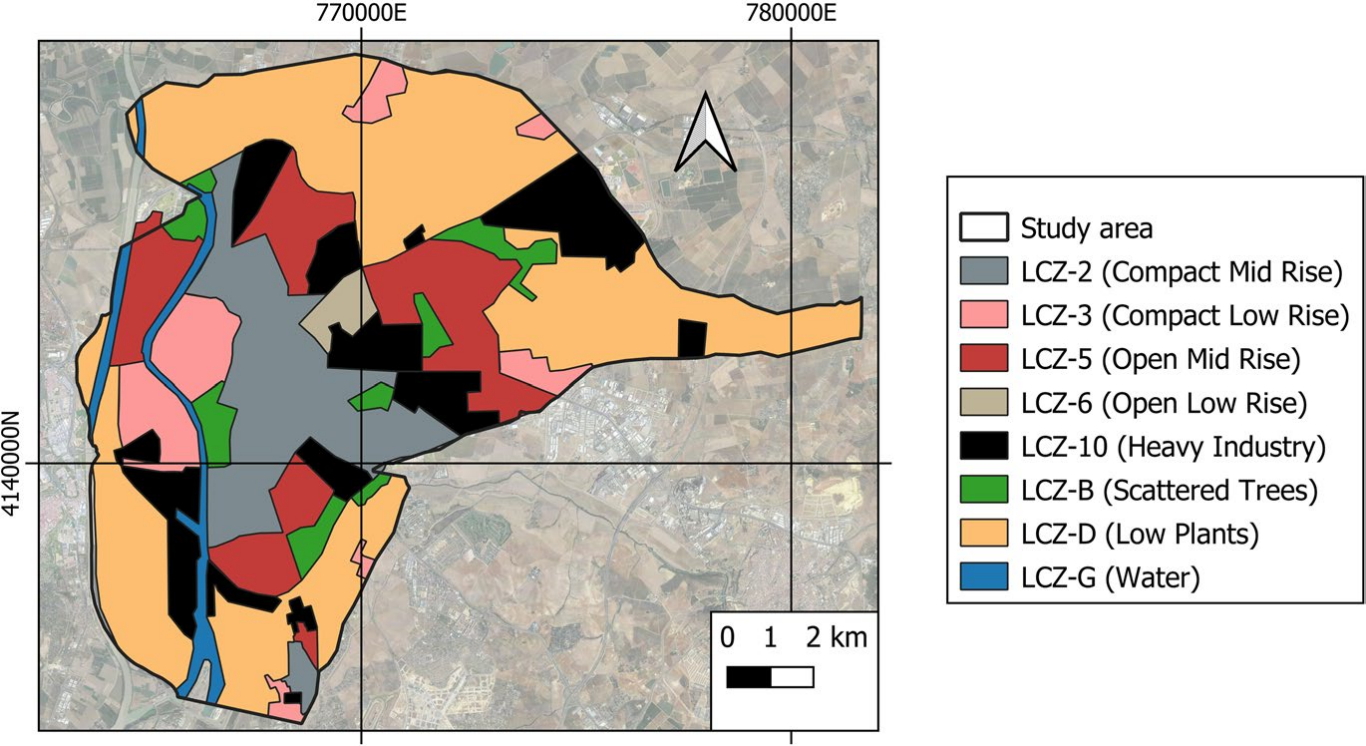
LCZ established for the city of Seville

As a result, each LCZ shares characteristics based on its shape and layout (Stewart & Oke, 2012) as long as those characteristics are maintained, allowing for extrapolation to similar urban areas elsewhere. The utility of this function is well documented in many landscape characterization studies (Anjos et al., 2020; Brousse et al., 2019; Emmanuel & Krüger, 2012; Equere et al., 2020; Khamchiangta & Dhakal, 2019; Wang & Ouyang, 2017). Following Stewart and Oke’s (2012) models and criteria, the resulting LCZ can be seen in Fig. 3, which includes compact mid and low rise (2 and 3), open mid and low rise (5 and 6), heavy industrial (10), low plants (LCZ-d), scattered trees (LCZ-B), and water (LCZ-G). Figure 4 depicts the classification of the zones.

The steps carried out for LCZ identification were as follows: (1) analysis and study of the city based on images from Google Earth and Google Street View (Yang et al., 2019); (2) identification of the orientation parameters and urban morphology of the images of the previous point; (3) delimitation of the different LCZs based on the construction characteristics of the studied areas (height of buildings, green spaces, road areas); (4) comparison of the resulting LCZ map with Google Earth images. A 95% match was found in a comparison of LCZ plans and Google images using 200 benchmarks. Only ten points did not reflect LCZ similarity. These, however, were manually corrected.

### Sentinel 2 images

Sentinel 2 images the earth’s surface in a multispectral mode. Changes in the earth’s surface and vegetation can thus be tracked from anywhere on the planet. The images are made up of 12 spectral bands with a resolution of 10 to 60 m. The images were obtained from the European Space Agency (ESA) via the Copernicus Open Access Hub. For this study, all level 2 images available for the months of July and August 2017 with a cloudiness index of less than 5% were chosen (07/26; 07/31; 08/05; 08/15; and 20/08). The presence of clouds in Sentinel images is typically a major issue due to pixel and information loss. A cloud index of less than 5% is sufficient to ensure that any potential loss of information has no effect on the results. The mean values of the five images were obtained using these images in order to obtain the various indices. Following download, the images were orthorectified and atmospherically corrected using the Sentinel Toolbox (S3TBX) in the SNAP software. Through the aerosol optical thickness (AOT) factor, this process works on band 10 to correct for the presence of clouds and on the blue, red, and SWIR bands to improve atmospheric transparency. The georeferencing used after this process was ETRS89/UTM Zone 29N.

### Land use/land cover

The earth’s climate system depends heavily on the land surface temperature (LST), and there are numerous LST studies that use different land covers to determine a relationship between the two elements (Shafizadeh-Moghadam et al., 2020; Otukei and Blaschke, 2009; Amindin et al., 2021). Moreover, there are several validated LULC products, each with its own set of covers (Corine Land Cover). Our study focused on five different types of land uses: vegetation, bodies of water, buildings, bare soil, and farmland. As a result, it was decided to manually obtain the LULC. First, an image with red, green, and blue bands was created using Sentinel 2 images. The QGIS software and the SVM method were then used to create the LULC map. As a training system, the QGIS plugin dzetsaka was used, which allows soil classification by determining different zones based on each type of soil. This methodology was introduced in 1999, and its main advantage is that it uses fewer samples while achieving high precision (Amindin et al., 2021). Several studies have used this method to accurately determine land cover differentiation (Amindin et al., 2021; Karakuş, 2019; Otukei & Blaschke, 2010; Shafri & Ramle, 2009).

The precision of the obtained LULC planes was determined using a precision matrix (Campbell, 1996), which is widely used in investigations requiring land surface classification (Yoo et al., 2019). To do so, we began with a sample of 200 randomly selected points within the study area. They were subsequently divided into two parts: 75% (150 points) for training and 25% (50 points) for testing. We trained the model on training data first, and then applied it to the remaining data and evaluated its performance with the precision matrix.

### NDVI

The NDVI is calculated using Eq. 1 and the red (Red) and near-infrared (NIR) bands. NDVI values range from −1 (no vegetation) to 1 (leafy vegetation):

1 $$\textrm{NDVI}=\frac{\textrm{NIR}-\textrm{Red}}{\textrm{NIR}+\textrm{Red}}$$

Given the results obtained with Eq. 1, the PV can be calculated through Eq. 2 (Rajeshwari, 2014; Yu et al., 2014).

2 $$\textrm{PV}={\left[\frac{\textrm{NDVI}-{\textrm{NDVI}}_{\textrm{min}}}{{\textrm{NDVI}}_{\textrm{max}}-{\textrm{NDVI}}_{\textrm{min}}}\right]}^2$$

where NDVI min and NDVI max are the minimum and maximum values of the NDVI variable of each image used in the investigation and the NDVI is obtained from Eq. 1.

### NDBI

The NDBI displays the proportion of built-up area versus undeveloped land in each pixel of a satellite image. According to Eq. 3, it was calculated using shortwave infrared (SWIR) and near-infrared (NIR) bands (Zha et al., 2003):

3 $$\textrm{NDBI}=\frac{\textrm{NIR}-\textrm{SWIR}}{\textrm{NIR}+\textrm{SWIR}}$$

### UI

This index distinguishes built-up or under-construction areas from rural areas with vegetation or bare soil. According to Eq. 4, the UI allows us to identify large urban areas using the second band of shortwave infrared (SWIR2) and near-infrared (NIR) (Kawamura et al., 1996):

4 $$\textrm{UI}=\frac{{\textrm{SWIR}}_2-\textrm{NIR}}{{\textrm{SWIR}}_2+\textrm{NIR}}$$

### UrbClim model

The city’s ambient temperature and relative humidity data were obtained on the specified dates from the UrbClim model developed by VITO (Flemish Institute for Technological Research). This approach is used to generate urban climate data in European cities as part of the Copernicus Climate Change Service program, which was implemented by the European Center for Weather Forecasts (ECMWF). The large-scale model dataset is derived from the ERA-5 reanalysis and is then reduced to the urban agglomeration scale. The land uses in this model are provided by the CORINE LAND COVER, which is linked to a three-dimensional atmospheric module. This is a simple evaluation of the energy balance of the urban surface. It was designed to target the spatial scale of a city, to be quick and complete, and to produce results with high precision. It is based on the Land Surface Interaction Calculation (LAICA) transfer scheme between the atmosphere and the geosphere, which was later modified to include the surfaces of urban areas (De Ridder et al., 2015). Therefore, the UrbClim model uses the different covers from CORINE as input for the soil surface. In our research, this situation contrasts with the use of the different LCZs as previously indicated and justified. The UrbClim model has been validated in several European cities, including Madrid (Martí Ezpeleta & Royé, 2021), Antwerp, Bruges, and Ghent (Verdonck et al., 2018). The data from UrbClim on ambient temperature, relative humidity, and heat stress index were later linked to the LCZ map using the QGIS software which is necessary to obtain the results of the environmental variables for each LCZ.

### Processing and georeferencing (SNAP)

Using the SNAP software, the processing (geometric and radiometric calibration) and georeferencing were carried out. For this, the radiometric calibration process began, which consists of a process that allows for the correction of possible image pixel distortions. Following that, we proceeded with the geometric correction that allows us to correct the possible distortions of the sensor. Finally, the ETRS89/UTM zone 29N System was reprojected.

### Environmental data validation

The environmental temperature and humidity data obtained from the UrbClim model were later compared and validated with the data from the meteorological station located in Seville that is owned by the State Meteorological Agency (AEMET). This procedure is critical for validating the values obtained from the UrbClim model. The validation results for temperature and humidity were as follows: *R*^2^=0.95, RMSE=1.8, MBE=0.089 and *R*^2^=0.96, RMSE=4.5, and ME 0.89.

### Heat waves

The AEMET database was used to obtain data on the days, duration, and thermal anomalies (temperature exceedances above the 95th percentile values) of the heat waves. A heat wave, as defined by AEMET, is an episode of at least three consecutive days during which a minimum of 10% of the stations record maximums above the 95% percentage of their series of maximum daily temperatures for the months of July and August during the period of 1971–2000. Between July and August of 2017, the city of Seville experienced four heat wave episodes. The first occurred between July 12 and 16, with a 2.6 °C thermal anomaly. The second occurred between July 28 and 30, with a 3.9 °C thermal anomaly. The third occurred between August 2 and 6, with a 1.6 °C thermal anomaly. Finally, the fourth heat wave occurred between August 20 and 22, with a 2.9 °C thermal anomaly.

### Heat stress index

The heat index (HI) formula (Eq. 5), developed in 1990 (Rothfusz & Headquarters, 1990) and later modified (Brooke Anderson et al., 2013), was used to calculate the heat stress index:

5 $$\textrm{Heat}\ \textrm{Index}\ \left(\textrm{HI}\right)=-8.78469475556+\left(1.61139411\times T\right)+\left(2.33854883889\times H\right)-\left(0.14611605\times T\times H\right)-\left(0.012308094\times {T}^2\right)-\left(0.0164248277778\times {H}^2\right)\kern0.5em +\left(0.002211732\times {T}^2\times H\right)+\left(0.00072546\times T\times {H}^2\right)-\left(0.000003582\times {T}^2\times {H}^2\right)$$

According to this equation, HI is the heat stress index in °C, *T* is the air temperature in °C, and *H* is the relative humidity in %. Based on the results obtained (Table 1), the effects on the population can be calculated (Kotharkar et al., 2021; Stewart & Oke, 2012).

**Table 1:** Classification of heat indices and heat risk conditions

Heat index	Classification of heat	HI	General effect on people
HI-1	No risk	*< 26.00*	No risk to population group.
HI-2	Very warm	*26.66–32.21*	Fatigue possible with prolonged exposure and physical activity.
HI-3	Hot	*32.22–39.43*	Sunstroke, heat cramps, or heat exhaustion LIKELY and heat stroke POSSIBLE with prolonged exposure and/or physical activity.
HI-4	Very hot	*39.44–51.10*	Sunstroke, heat cramps, or heat exhaustion POSSIBLE with prolonged exposure and/or physical activity.
HI-5	Extremely hot	*>51.11*	Heat/sunstroke HIGHLY LIKELY with continued exposure.

Source: Kotharkar et al. (2021)

The column with the values in italics indicate the value in °C of the heat stress index associated with the first column

### Urban hot spots

The hot zones or spaces (UHS) can be located using the UrbClim model’s environmental temperature. Because of their high temperatures, they are classified as uninhabitable by the general public. Equation 6 can be used to identify the locations (Sharma et al., 2021):

6 $$T>\mu +2\ast \sigma$$

where *σ* and *μ* are respectively the standard deviation and mean values of the ambient temperature of the area in °C.

## Results

### Results of the NDVI, PV, NDBI, and UI indices

**Fig. 5:**
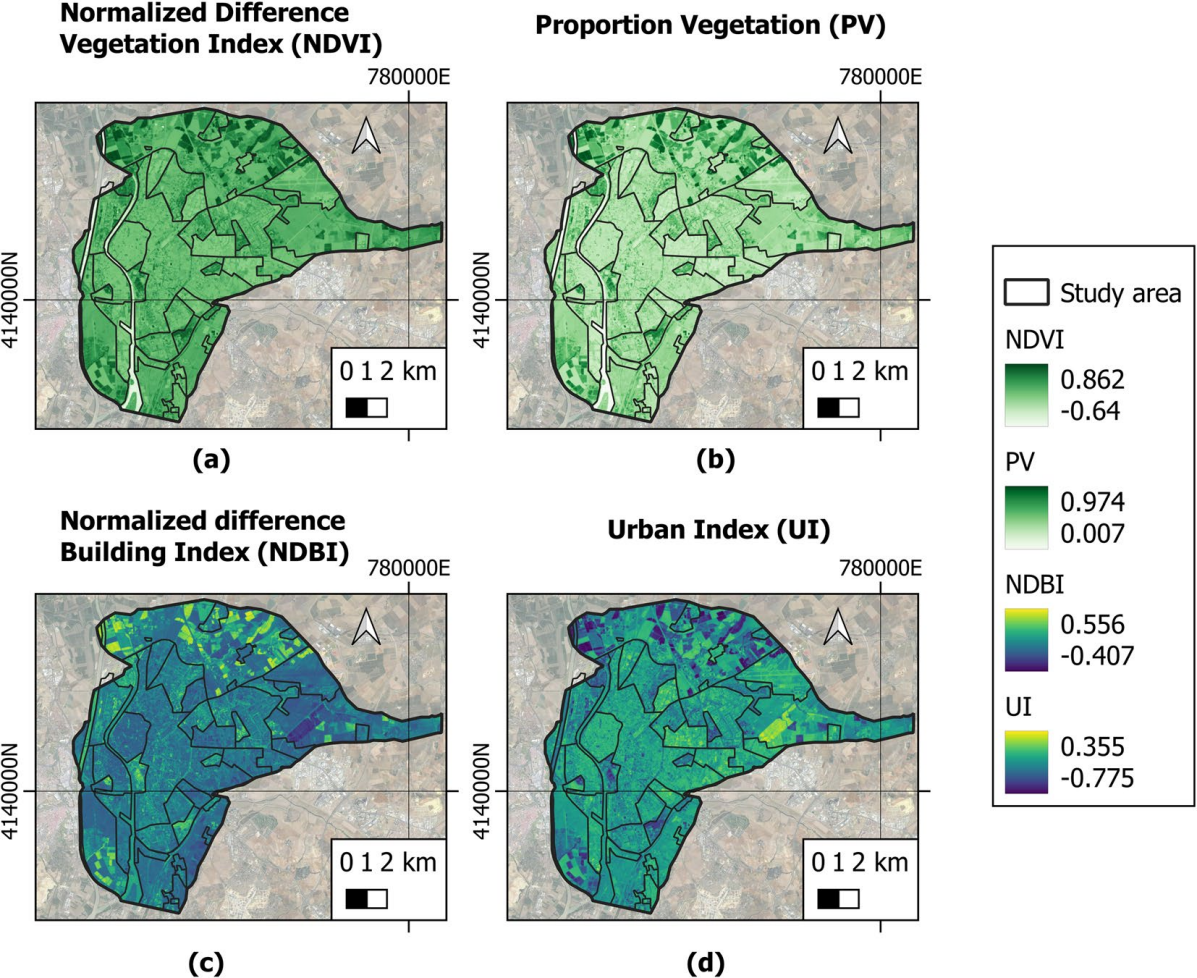
Indices **a** NDVI, **b** PV, **c** NDBI, and **d** UI of the area under study

The analysis of the NDVI, PV, NDBI, and UI indices of the area under study can be seen in Fig. 5.

The average values obtained for these indices in the city of Seville were as follows: NDVI 0.284, PV 0.385, NDBI 0.041, and PV 0.132. In general, these are typical values for a city with the overall characteristics of Seville. Given that the images are from the summer (July and August), and based on the NDVI and PV values, the vegetation in the study area would be classified as sparse. According to the NDBI and UI values, compact areas with medium density outnumber open areas with low density.

**Fig. 6:**
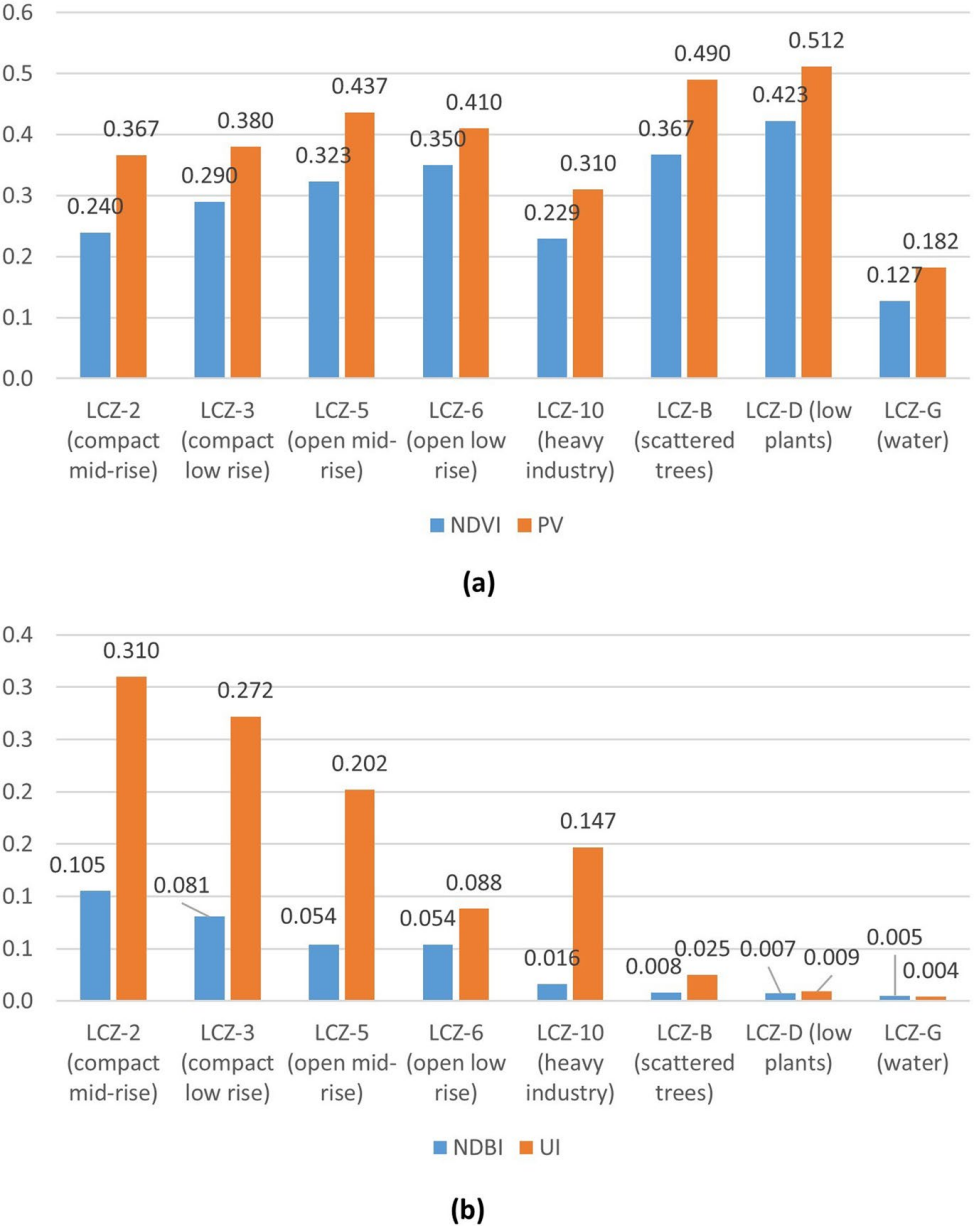
Indices **a** NDVI and PV, and **b** NDBI and UI of the area investigated by LCZ

Figure 6 displays the mean values of NDVI, PV, UI, and NDBI in each LCZ. The NDVI index presents the highest values for LCZ-D (low plants), B (scattered trees), 6 (open low rise), and 5 (open mid-rise), while the lowest values are seen for LCZ-G (water), 10 (heavy industry), 2 (compact mid-rise), and 3 (compact low rise). The PV index presents the highest values in LZC-D (low plants), B (scattered trees), 5 (open mid-rise), and 6 (open low rise), whereas the lowest values correspond to LCZ-G (water), 10 (heavy industry), 2 (compact mid-rise), and 3 (compact low rise). These values confirm that the vegetation is lusher in rural areas and in open areas as opposed to the industrial and compact areas of the city.

The NDBI index presents the highest values in LCZ-2 (compact mid-rise), 3 (compact low rise), 5 (open mid-rise), and 6 (open low rise). The lowest values are obtained in LCZ-G (water), D (low plants), B (scattered trees), and 10 (heavy industry). The PV index presents the highest values in LZC-2 (compact mid-rise), 3 (compact low rise), 5 (open mid-rise), and 10 (heavy industry); the lowest values are in LCZ-G (water), D (low plants), B (scattered trees), and 6 (open low rise). Such findings indicate that urban areas have higher occupancy and density than rural areas, and that industrial areas are more compact (when compared to open areas).

### Spatio-temporal evaluation of the LULC

**Fig. 7:**
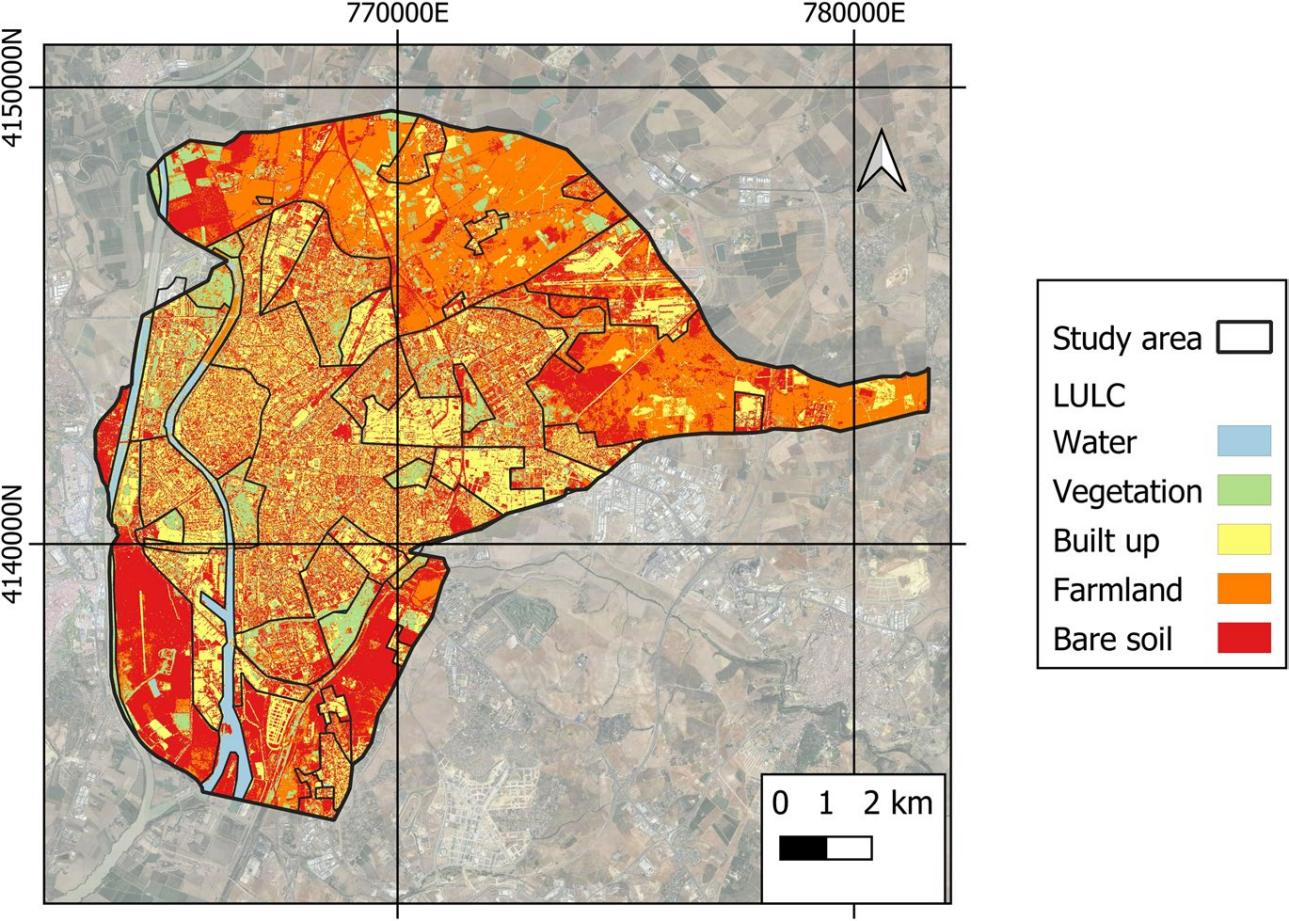
LULC coverage of the area under study

Figure 7 depicts an analysis of the various LULC coverages in the LCZ. In terms of average values, the coverage with the greatest area in Seville is built-up (39.05%), while the coverage with the smallest area is water (2.31%). Vegetation coverage (10.96%), bare soil (18.46%), and farmland (29.22%) have intermediate values.

**Fig. 8:**
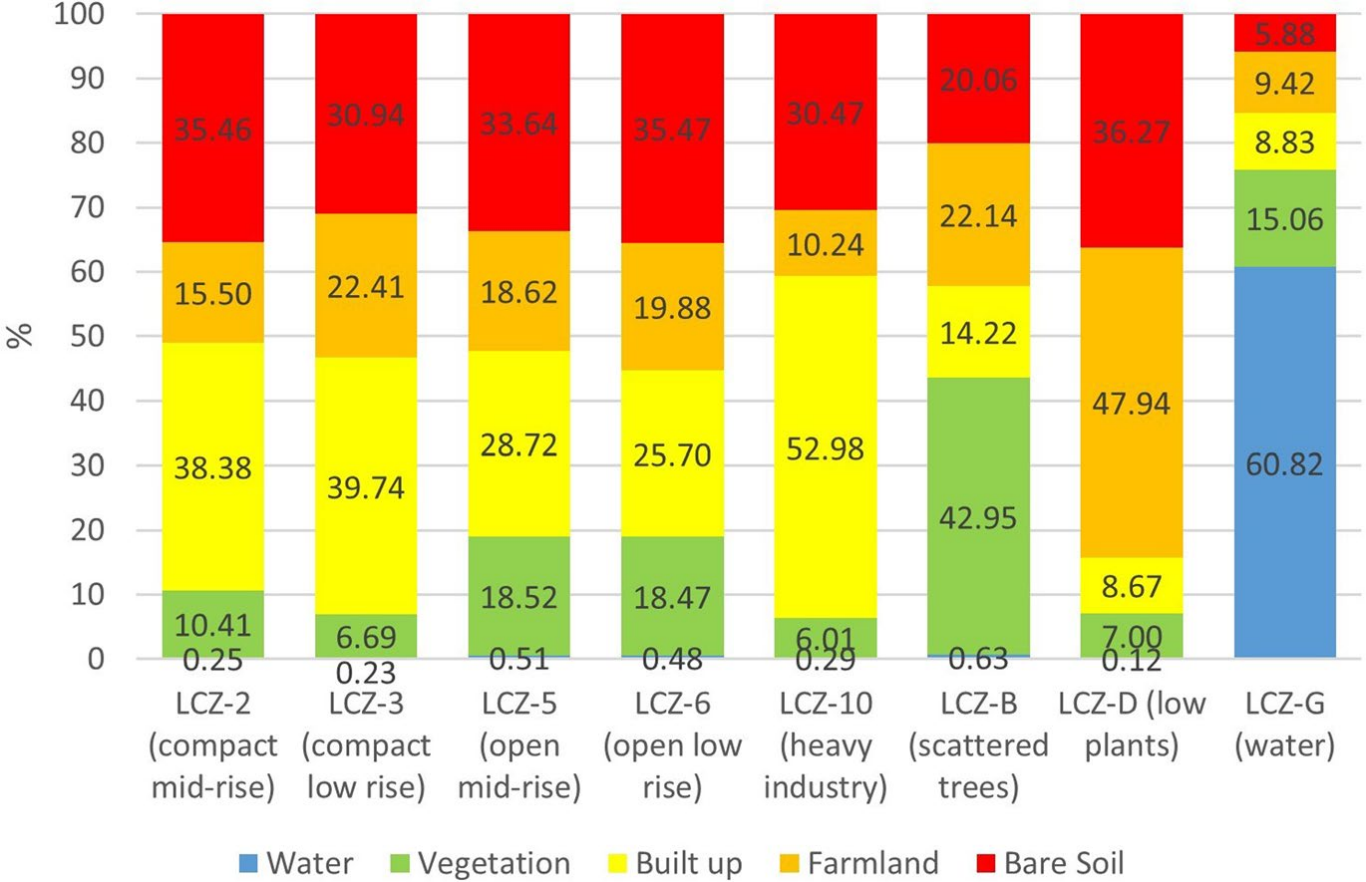
Mean LULC values in the different LCZs

Figure 8 depicts LCZ’s various LULC coverages. These values of the various LULC coverages identified within each LCZ will allow us to confirm the results obtained with the NDVI, PV, NDBI, and UI indices. The greatest built-up coverage is found in LCZ-10 (heavy industry), 2 (compact mid-rise), and 3 (compact low rise). To the contrary, the greatest vegetation coverage is found in LZC-B (scattered trees). Farmland coverage is mostly found in LZC-D (low plants).

It is important to note how the compact areas of the city (LCZ-2 compact mid-rise and 3 compact low rise) present an average built-up coverage (39.06%) greater than the open areas (27.21%) (LCZ-5 open mid-rise and 6 open low rise). The average vegetation cover is also higher in open areas (18.49%) than in compact areas (8.51%). The coverage results determined here for the city of Seville, however, are adequate in the context of the Mediterranean Sea basin, according to the Köppen-Geiger classification of typical vegetation and adapted crops. The precision matrix with 200 randomly selected points produced an accuracy of 82% for the various LULCs with a 95% confidence interval. The Tau coefficient was 0.811, while the Kappa coefficient was 0.921. A manual correction was performed after the precision process to improve the precision of the obtained LULC plan.

### Spatio-temporal evaluation of temperature

**Fig. 9:**
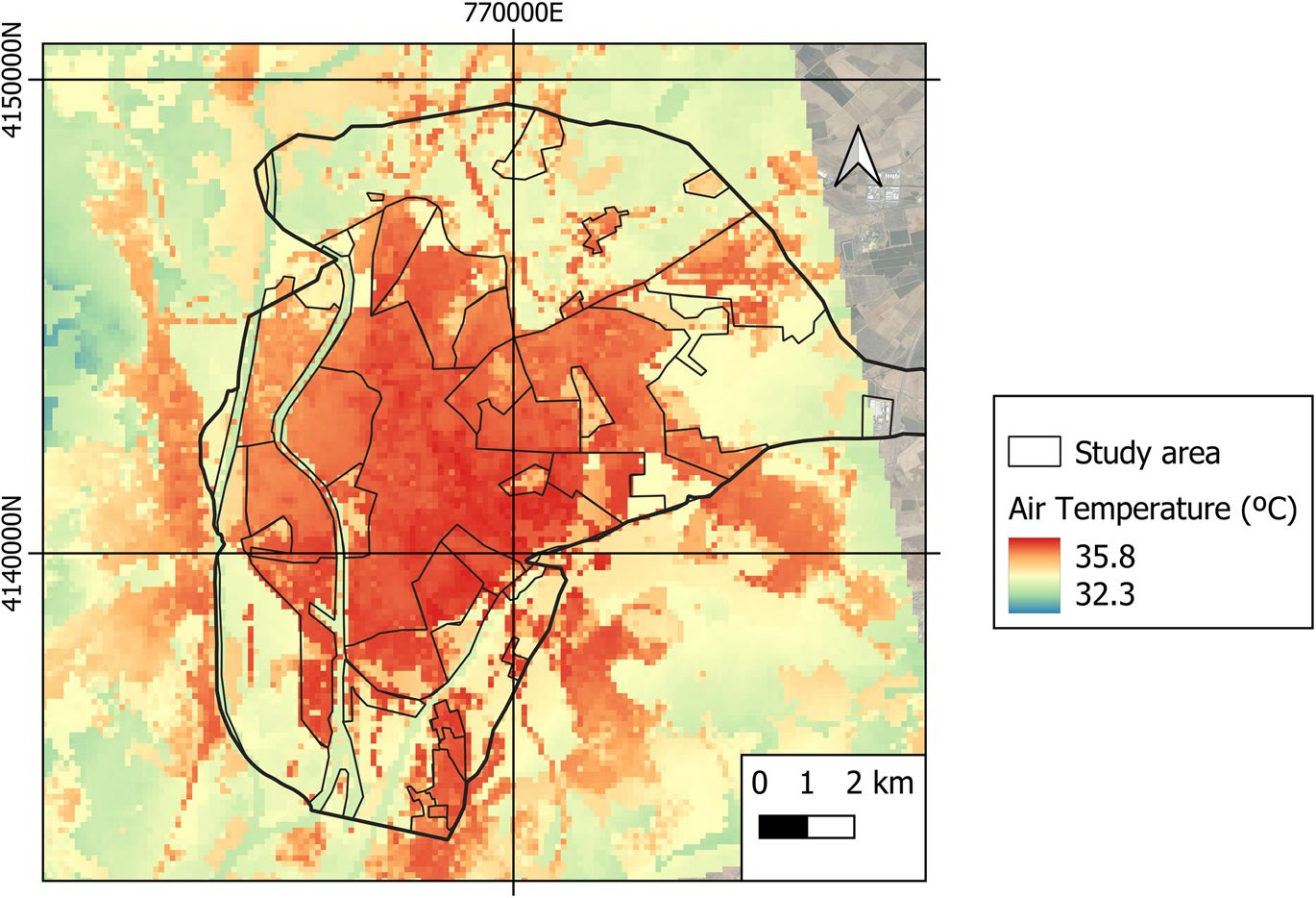
Average air temperature in the city of Seville throughout the study period

Figure 9 reflects the average air temperature in the city of Seville on a daily basis during the study period.

**Fig. 10:**
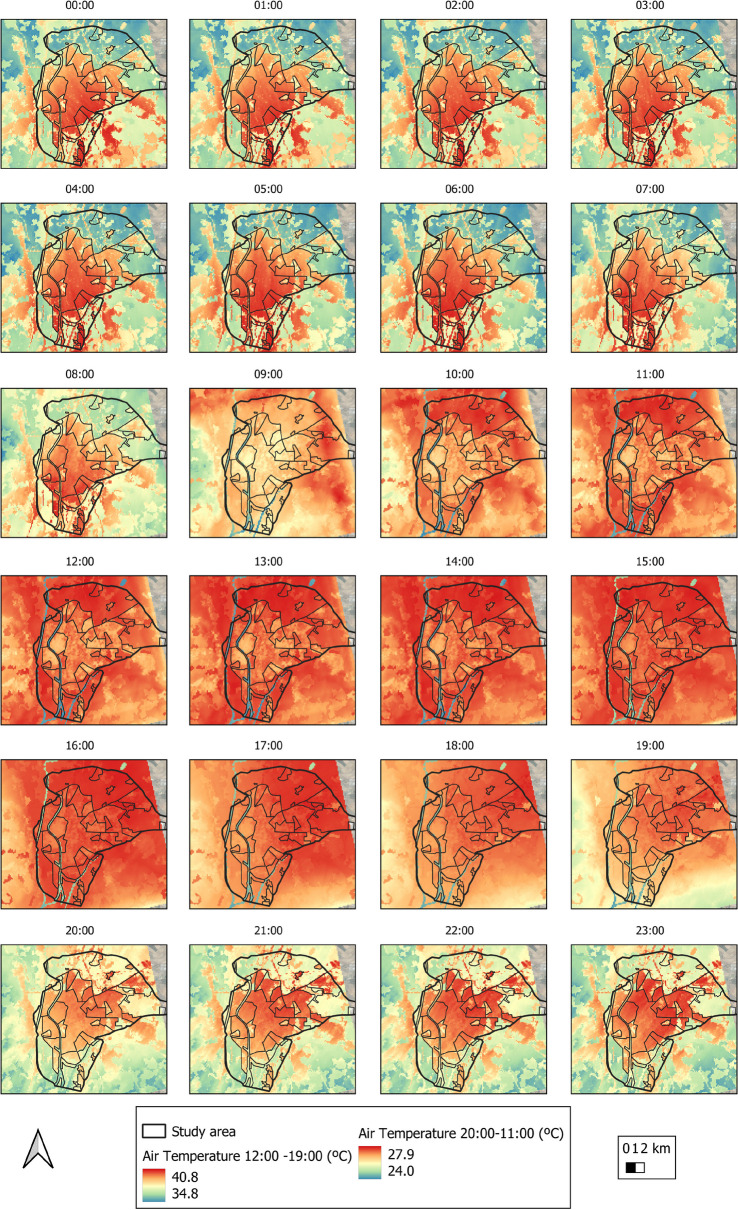
Evolution of average temperatures per hour throughout the study period

The average temperature was 31.8 °C throughout the study period (the average maximum and minimum temperatures ranged between 34.1 and 29.9 °C). The highest temperatures (higher NDBI and UI indices) are concentrated in urban areas. The lowest values are found in rural or suburban areas (higher NDVI and PV indices). Figure 10 depicts the evolution of average temperatures over the course of the study. The highest values are obtained between 09:00 and 20:00, with rural areas having higher values. This is because, during the day, the solar radiation received in rural areas is greater than in urban areas due to the shade generated by trees and buildings. The shade generated by trees and buildings prevents radiation from reaching the ground, thus heating enclosures and impermeable surfaces (Li & Meng, 2018). In addition, urban areas have higher thermal inertia, resulting in slower heating rates than rural areas. Another important factor to consider is the cooling rate of vegetated areas (Arbuthnott & Hajat, 2017; Yang et al., 2020). Between 8:00 p.m. and 9:00 a.m., the city has the highest temperatures, while rural areas have lower values. When the sun sets, urban areas remain hot, whereas rural areas cool quickly. Because high thermal absorption materials are used in urban construction, they release heat into the atmosphere/environment at night (Dwivedi & Mohan, 2018; Hidalgo & Arco, 2021; Santamouris, 2020), resulting in the urban heat island (UHI) phenomenon.

**Fig. 11:**
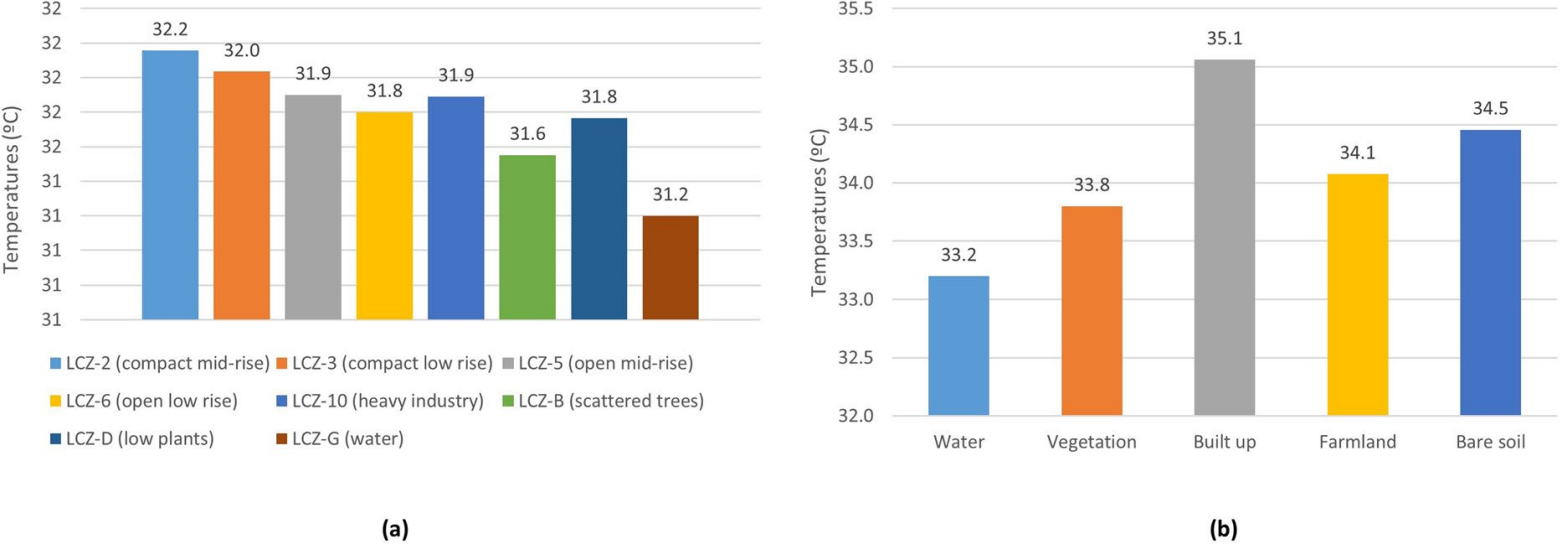
Ambient temperature of **a** LCZ and **b** LULC of the study object area

Figure 11a shows the average temperature for each LCZ. The average temperature is higher in LCZ-2 (compact mid-rise), 3 (compact low rise), 10 (heavy industry), and 6 (open low rise). The lowest average temperatures correspond to LCZ-G, D, B, and 5. Thus, the average temperature in urban areas is higher (31.96 °C) than in rural areas (31.53 °C). Within urban areas, the compact LCZs (32.1 °C) present higher temperatures than the open LCZs (31.9 °C). Figure 11b displays the mean temperatures by LULC. The highest temperatures correspond to built-up (35.1 °C) and bare soil (34.5 °C) coverage; the lowest temperatures pertain to water (33.2 °C) and vegetation (33.8 °C) coverage.

### Spatio-temporal evaluation of humidity

**Fig. 12:**
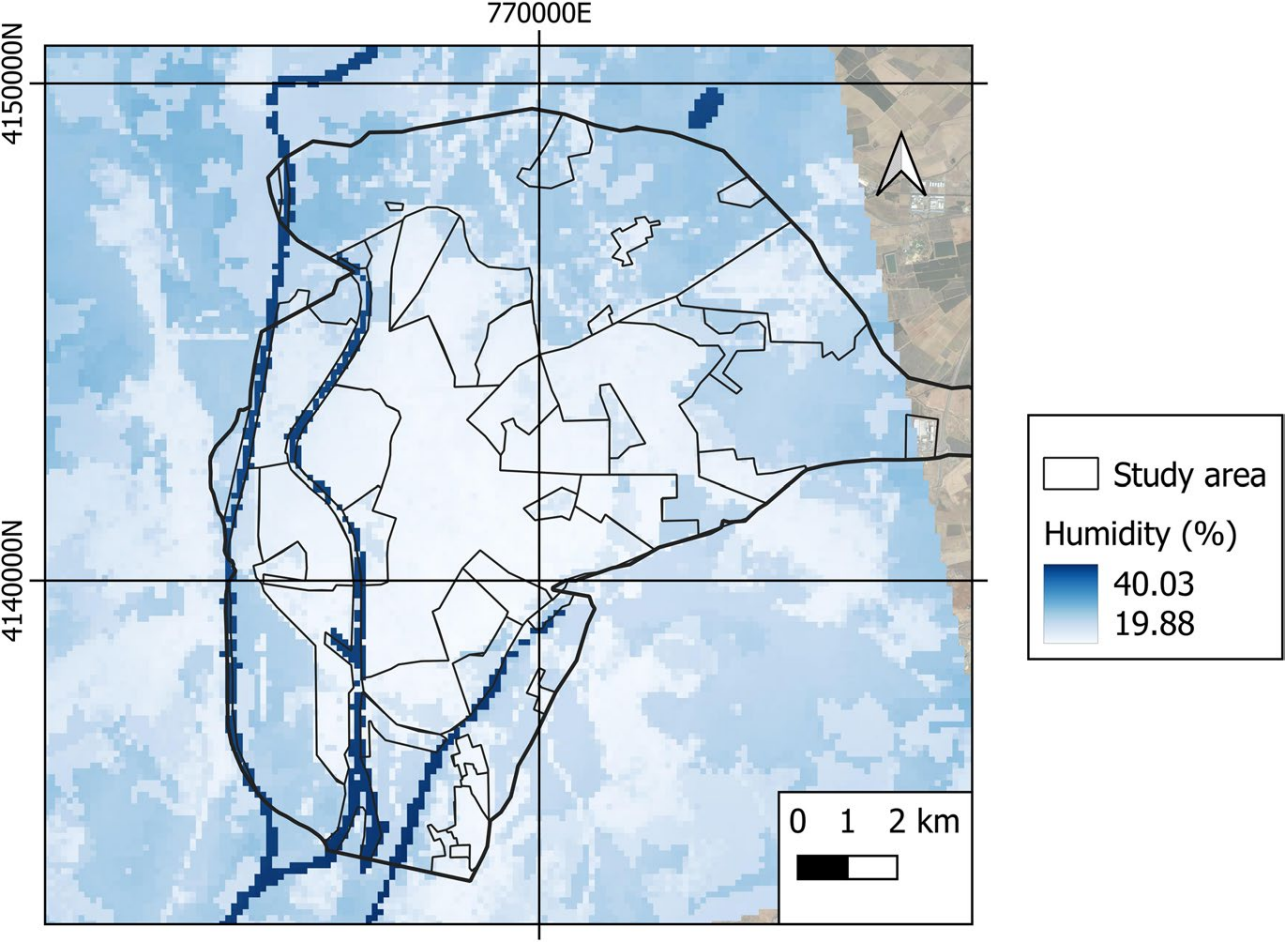
Average relative humidity of the air in the city of Seville during the entire study period

Figure 12 depicts an analysis of the average relative humidity in Seville over the entire study period.

**Fig. 13:**
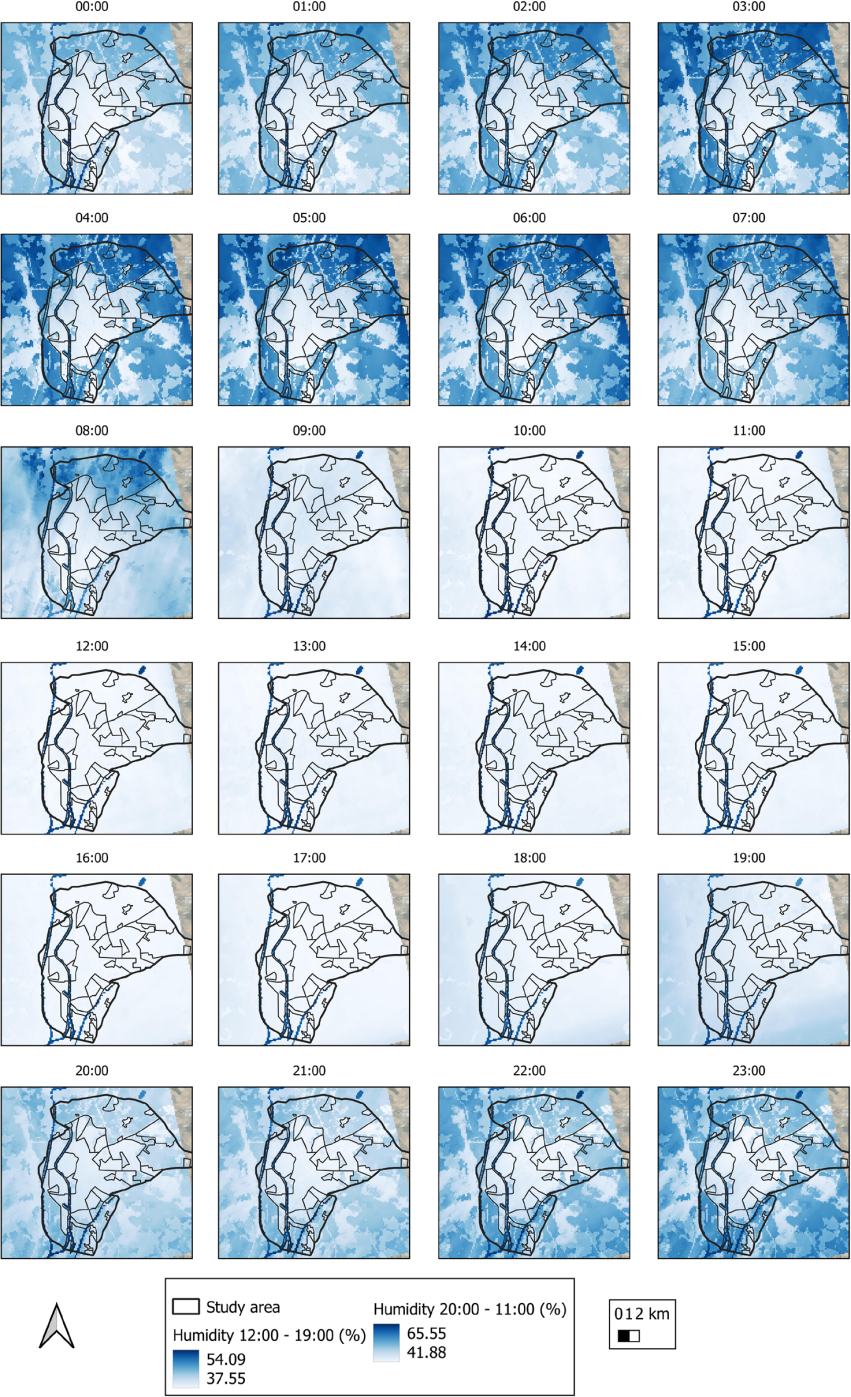
Space-time variability of relative humidity in the city of Seville

Throughout the entire study period, the average humidity was 32.4%, while the maximum and minimum mean values were 40.1% and 19.9%, respectively. The highest humidity was seen for rural areas (the lowest values pertaining to urban areas). Figure 13 depicts the evolution of relative humidity over time. The lowest values are obtained between 09:00 and 19:00, with rural areas having lower values than urban areas. This is due to plant evapotranspiration, which releases some of its moisture into the atmosphere at high temperatures, favoring environmental cooling (Gago et al., 2013; Solecki et al., 2005), and the effect produced by temperature, that is, the lower the temperature, the higher the relative humidity as the air approaches the degree of saturation.

**Fig. 14:**
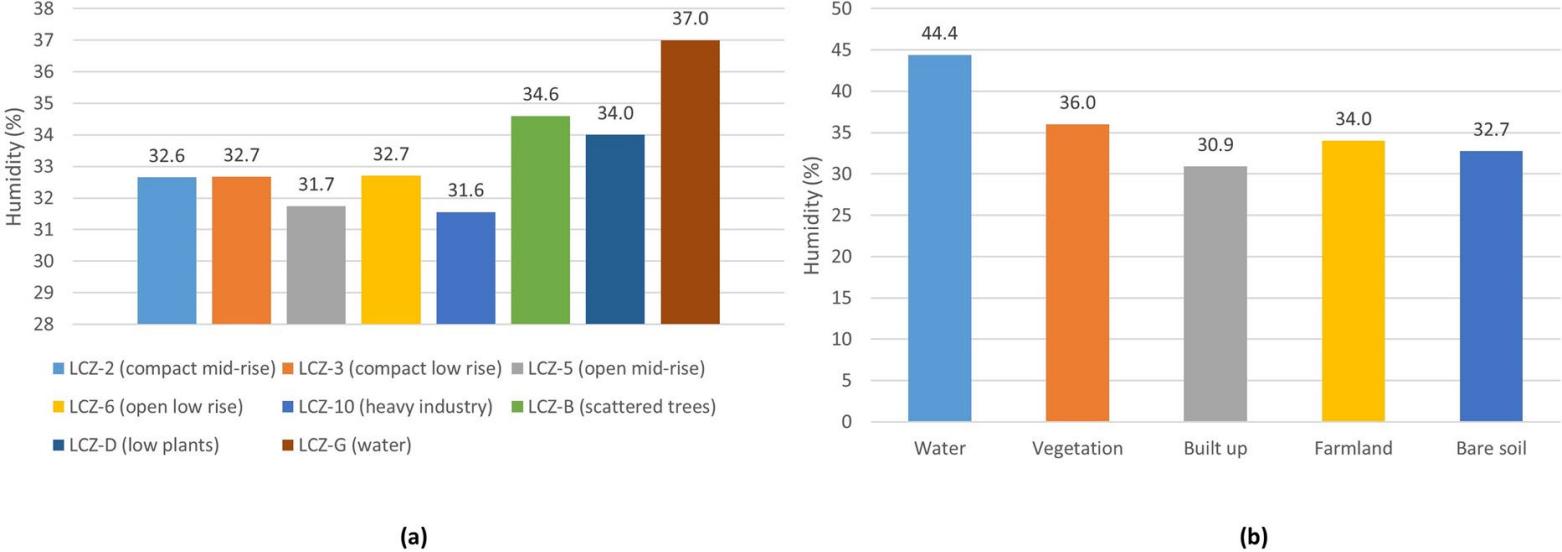
Relative humidity of **a** LZC and **b** LULC of the study object area

Figure 14a depicts the average relative humidity for the various LCZs. The average relative humidity in LCZ-D (low plants), B (scattered trees), and G (water) is the highest. The areas having the lowest average relative humidity are LCZ-10, 5, and 2. The average relative humidity by LULC is shown in Fig. 14b. It is higher in water cover (44.4%) and vegetation (36%), and lower in built-up (30.9%) and bare soil (32.7%) covers. As a result, the LCZ and LULC with higher values for vegetation and green areas also have higher humidity values; areas and coverages with greater building expanses have lower humidity values.

### Heat stress index

**Fig. 15:**
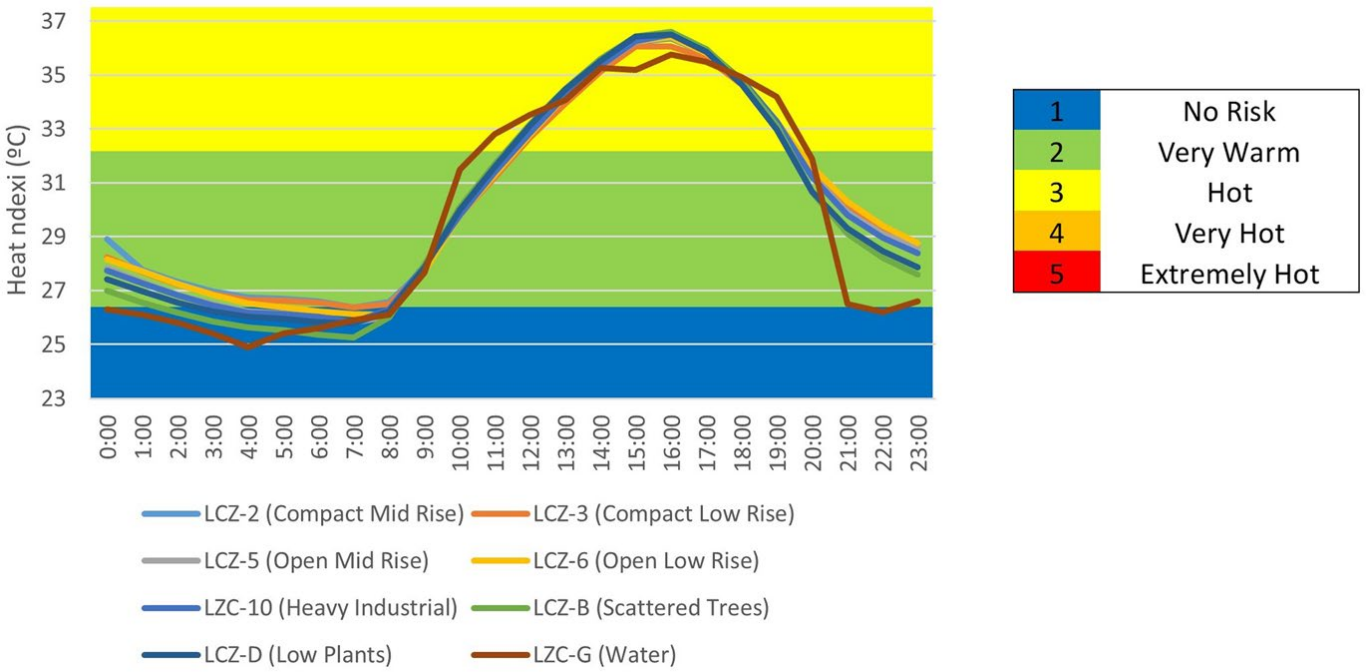
HI index under normal environmental conditions by LCZ

Figure 15 shows the HI results for Seville as classified by LCZ.

**Fig. 16:**
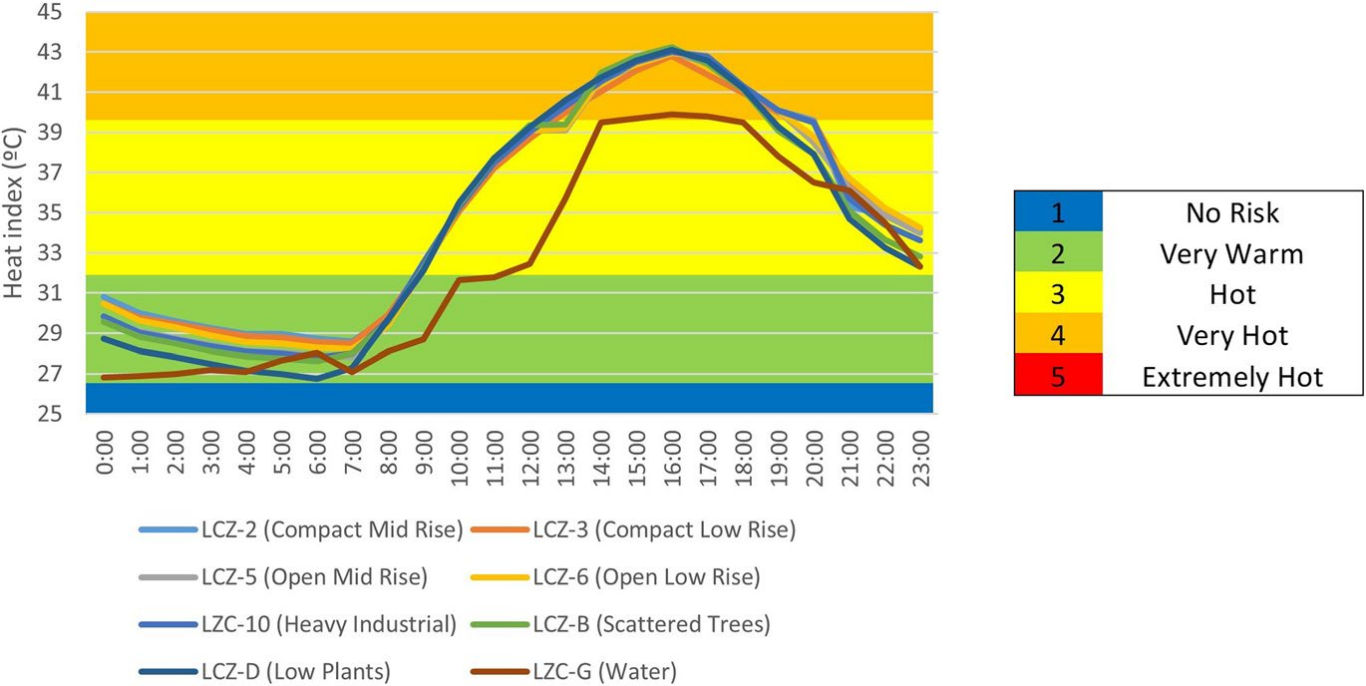
HI index in periods of heat wave by LCZ

In general, the city has a “no risk” HI between 10:00 p.m. and 9:00 a.m., but it rises to very warm from 10:00 a.m. to 11:00 a.m. and 8:00 p.m. to 9:00 p.m., reaching a maximum HI of heat from 12:00 to 19:00. Rural areas have a higher HI value in the morning than urban areas. On the contrary, rural areas have lower HI values in the afternoon than urban areas, which need time to reduce the value of HI. Figure 16 depicts the HI results obtained and qualified by LCZ during the heat wave periods. The greater the heat, the greater the intensification of the HI in all LCZs, such that the HI is classified as very warm from 00:00 to 09:00, and then intensified to hot from 10:00 to 12:00 h and 8:00 p.m. and 11:00 p.m.; and the HI reaches its maximum value, very hot, from 1:00 p.m. to 7:00 p.m. We can see that urban areas increase their HI value faster than rural areas in the mornings, but drop more slowly in the afternoons. Meanwhile, the compact zones (LCZ-2 compact mid-rise and 3 compact low rise) and industrial zones (LCZ-10 heavy industry) show less resistance to the HI index—they rise faster in the mornings and fall slower in the afternoons/evenings.

**Fig. 17:**
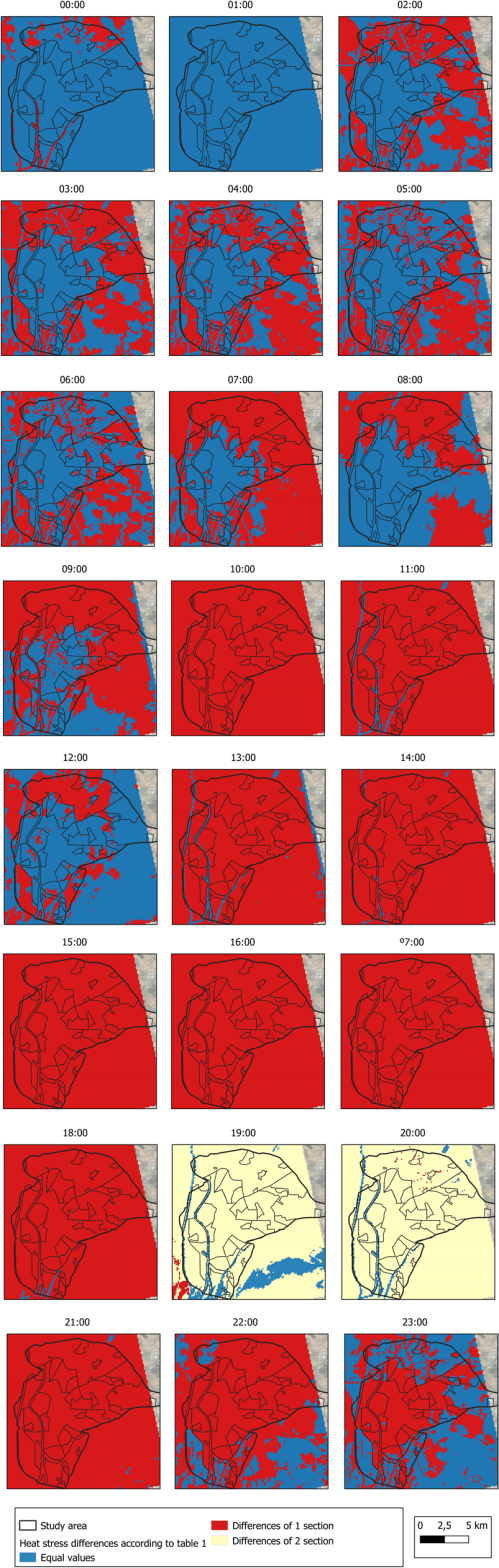
Differences in HI between conditions (normal environmental conditions and under heat wave conditions) by LCZ as scored in Table 1

Figure 17, using the stress index scale established in Table 1, reports the differences in HI between the two environmental conditions studied (normal environmental conditions and heat wave conditions). As a result, the blue color indicates no differences between the two climatic conditions, the red color indicates 1-level intensification under heat wave conditions, and the yellow color indicates 2-level intensification. The most noticeable differences occur between 10:00 a.m. and 9:00 p.m. In the vast majority of LCZ, there are two peaks on the HI scale between these periods.

### UHS identification

**Fig. 18:**
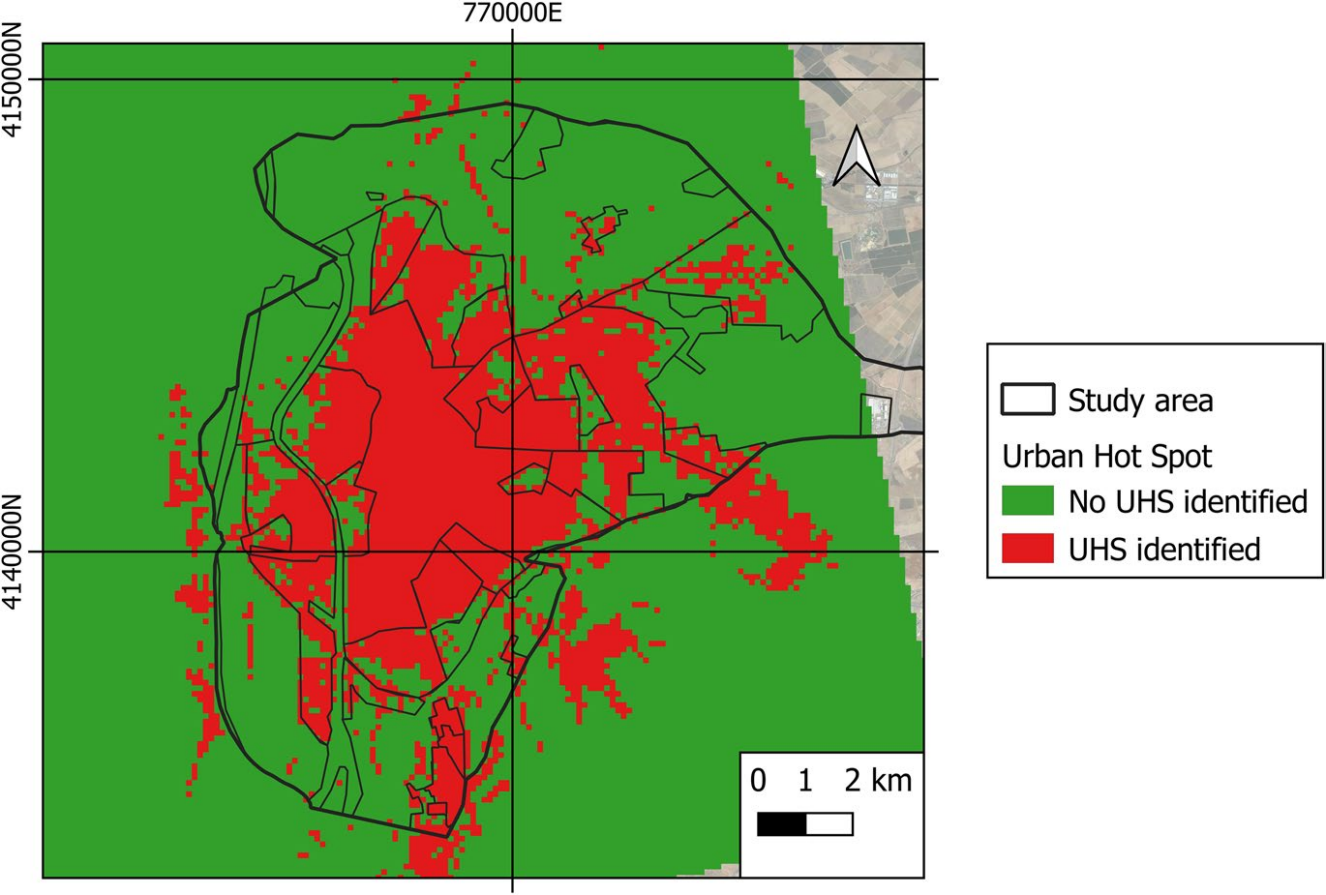
UHS of the area under study

Figure 18 reflects the spatio-temporal analysis of the average UHS of the city of Seville. The areas classified as UHS occupy 35.95% of the total area. As seen, most of the UHS are located in urban areas—not rural areas, where there are hardly any spaces that qualify as UHS.

**Fig. 19:**
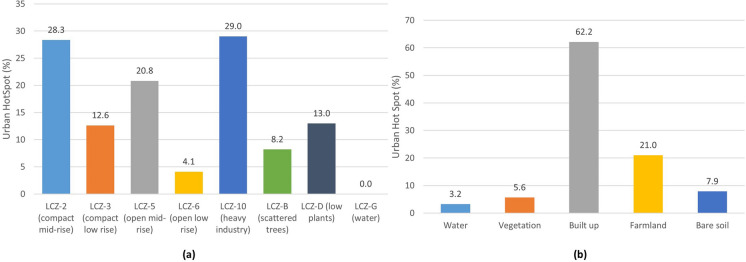
UHS (%) of the entire period under study by different **a** LCZs and **b** LULCs

Figure 19a shows the occupancy percentage of the UHS in the different LCZs, while Fig. 19b highlights the occupancy rate in the different LULCs. It is clear that most of the areas classified as UHS are in LCZ-10 (heavy industry), 2 (compact mid-rise), and 5 (open mid-rise). In turn, LCZ-G (water), 6 (open low rise), and D (low plants) present fewer UHS spaces. In terms of LULC coverage, the one with the highest percentage of UHS is built-up, while water and vegetation have the lowest occupancy.

### Statistical analysis

Two complementary methods were used for our research’s statistical analysis: ANOVA and panel data. Both methods are appropriate for studying cross-sectional data in time series to confirm the existence of a trend and the factors that influence it. When there are different groups, the ANOVA is used to compare the variances between the means. It is typically used to see if there is a difference in the means of different groups. Panel data, on the other side, is often cited in the literature and involves the use of multiple regression models (Alcock et al., 2015; Chen et al., 2011; Fang & Tian, 2020) which allows for the inclusion of a larger amount of data than traditional methods. The phases to follow are the following (Chen et al., 2011): (1) determine if the effects of the analysis are fixed or random, and (2) evaluation of the model through the Wooldridge and Wald tests. For our investigation and after carrying out the indicated tests, the method according to Eq. 7 was used:

7 $${Y}_{it}=\beta\ {X}_{it}+\left({\alpha}_i+{\mu}_{it}\right),$$

where *μ*_*it*_ is the error of the model, *α*_*i*_ represents the individual effects, *X*_*it*_ are explanatory variables, *β* is an independent variable, 𝑡=time, and 𝑖=individual.

The results of the ANOVA test carried out on the NDVI, PV, NDBI, and UI indices and temperatures and humidity and reflected through the Shapiro-Wilk test indicate non-normal distributions within the different LCZs, since *p* value < 0.05. Therefore, to continue with the ANOVA analysis for non-normal distributions, it is necessary to perform the Kruskal-Wallis test, whose results are presented in Table 2.

**Table 2:** ANOVA test results for NDVI, PV, NDBI, and PV indices in the LCZ

Source	NDVI	PV	NDBI	UI	Temperature	Humidity	HI (1)	HI (2)
Difference of square	0.0001***	0.0001***	0.018*	0.0008***	0.0001***	0.0001***	0.0001***	0.0001***
*R*^2^	35.23	35.23	16.83	24.95	62.78	63.50	78.97	53.27

***p<0.001 and *p<0.05. *R*^*2*^, linear regression coefficient; *HI (1)*, HI without heat wave; *HI (2)*, Hi with heat wave

According to the above results, the NDVI, PV, and UI indices, temperature, humidity, and HI without and with heat wave give statistically significant relationships above 99% in the different LCZs, while the NDBI variable has a relationship of 95%.

The ANOVA test carried out on the different LULCs and UHS, as reflected by the Shapiro-Wilk test, signaled a normal distribution within the different LCZs (*p* > 0.05). The results (Table 3) attest that there are statistically significant differences above 99% between the LULC and the LCZ studied, which is supported by the *F* statistic> 0 and the variable Prob>chi^2^ < 0.001.

**Table 3:** ANOVA test results between LULC and LCZ

Source	LULC	UHS
Sum of square	29.1682	10.965
Mean of square	4.1668	1.5664
*F*	7.53	8.82
Prob>chi^2^	0.000***	0.000***
*R*^2^	0.235	0.2686

***p<0.001. *R*^*2*^, linear regression coefficient; *F*, *F* statistic

Accordingly, the HI values present statistically significant relationships that are greater than 99% between the different LCZs. The panel data was used to calculate the relationships between the indices (PV, NDVI, UI, LULC, and NDBI) and the dependent variable HI. The correlation coefficient was also calculated; then, applying the Generalized Least Squares (GLS) method, a regression was obtained and data panel analysis was carried out, giving the results indicated in Tables 4 and 5.

**Table 4:** Correlation coefficient between heat stress and the different indices

	HI	NDVI	PV	NDBI	UI	LULC
HI	1					
NDVI	−0.367	1				
PV	−0.376	0.979	1			
NDBI	0.185	0.678	0.778	1		
UI	0.258	−0.782	−0.857	−0.948	1	
LULC	−0.389	0.252	0.211	−0.137	0.056	1

**Table 5:** Data panel results between HI and indices

	*β*	*ρ* values	SD
NDVI	−2.378	0.038*	1.134
PV	−4.995	0.004**	1.686
NDBI	2.114	0.018*	0.884
UI	0.538	*0.483*	0.765
LULC	−0.168	0.001**	0.389
	*R*^2^=0.19	*F=8.81*	Prob>chi^2^= 0.000

**p<0.01 and *p<0.05. *sd*, standard deviation; *β*, coefficient; *R*^*2*^, coefficient of determination; *F*, *F* statistic

Table 4 shows how the HI presents a positive correlation with the NDBI (0.185) and UI (0.258) indices and a negative correlation with the NDVI (−0.367), PV (−0.376), and LULC (−0.389) indices.

The statistical analysis (Table 5) carried out at a confidence level of 95% and taking into account the results obtained from the *p* value found a significant and positive relationship of 95% between HI and NDBI. Additionally, there was a negative link with the PV and LULC variables of 99% and 95% with the NDVI variable. All of them were taking into account a confidence level of 95%. An adequate relationship is observed between the variables in terms of the values of *R*^2^, *F*, and Prob>chi^2^. As Prob>chi^2^ = 0.000, it can be affirmed that the fit is greater than 99%.

The statistical analysis was repeated for the heat wave periods undergone in Seville. Table 6 shows that the HI has a positive correlation with the NDBI (0.097) and UI (0.207) indices, yet a negative correlation with the NDVI (−0.403), PV (−0.388), and LULC (−0.447) indices. Building-related indices (NDBI and UI) show a lower correlation in heat wave conditions than in normal environmental conditions. However, the indices related to vegetation and LULC present a higher correlation under heat wave conditions.

**Table 6:** Correlation coefficient between HI and indices

	HI	NDVI	PV	NDBI	UI	LULC
HI	1					
NDVI	−0.403	1				
PV	−0.388	0.979	1			
NDBI	0.097	0.559	0.691	1		
UI	0.207	−0.732	−0.825	−0.934	1	
LULC	−0.447	0.197	0.151	−0.253	0.134	1

Statistical analysis (Table 7) reports a significant and positive relationship of 95% between the variable HI and NDBI, negative and over 99% with the LULC variable, and of 95% with the PV variable. A good relationship is seen between the variables when observing the values of *F*, *R*^2^, and Prob>chi^2^. As Prob>chi^2^ = 0.000, we can state that the adjustment level is greater than 99%.

**Table 7:** Data panel results between HI and indices

	*β*	*ρ*	SD
NDVI	−1.667	*0.138*	1.118
PV	−3.684	0.017*	1.521
NDBI	1.786	0.023*	0.777
UI	0.393	*0.563*	0.678
LULC	−0.170	0.000***	0.038
	*R*^2^=0.22	*F=10.15*	Prob>chi^2^= 0.000

***p<0.001, **p<0.01, and *p<0.05. *sd*, standard deviation; *β*, coefficient; *R*^*2*^, linear regression coefficient; *F*, *F* statistic

Following that, statistical analysis using the panel data method was used to determine the relationships between the HI and the UHS. Following the determination of the Pearson correlation coefficient, the regression was derived using the Generalized Least Squares (GLS) method, and data panel analysis was performed, yielding the results shown in Tables 8 and 9.

**Table 8:** Pearson Hi and UHS correlation coefficient

	HI	UHS
HI	1	
UHS	0.623	1

**Table 9:** Data panel results between Hi and UHS

	*β*	*ρ*	SD
UHS	0.545	0.000***	0.564
	*R*^2^=0.39	*F=93.39*	Prob>chi^2^= 0.000

***p<0.001. *β*, coefficient; *SD*, standard deviation; *R*^*2*^, linear regression coefficient; *F*, statistical

Table 8 shows how HI presents a positive correlation with the UHS variable (0.623).

From the statistical analysis (Table 9), a positive relationship greater than 99% is reported between the HI variable and the UHS. It is observed that there is a good relationship between the variables in terms of the values of *F*, *R*^2^, and Prob>chi^2^. Because Prob>chi^2^ = 0.000, it may be affirmed that the adjustment level is over 99%.

## Discussion

The vegetation-related NDVI and PV indices are known to have higher values in rural areas (LCZ-D, B, and G) and open urban areas (LCZ-5 and 6), as opposed to urban areas or compact and industrial areas (LCZ-2, 3, and 10). In contrast, it has been demonstrated that the NDBI and UI indices related to building have higher values in urban areas (open, compact, and industrial; LCZ-2, 3, 5, 6, and 10) and lower values in rural areas (LCZ-G, B, and D). As a result, the higher an area’s building density, the higher the NDBI and UI indices, and the lower the NDVI and PV indices. According to the LULC results, built-up coverage predominates in urban areas, while bare soil and farmland predominate in rural areas. The coverage of vegetation is found to be greater in rural areas than in urban areas, and virtually non-existent in industrial areas. This coverage is greater in open city areas than in compact areas. Such findings summarize the urban morphology of each LCZ studied and are consistent with similar studies conducted in other cities and territories (Avdan & Jovanovska, 2016; Diallo-Dudek et al., 2015; Hidalgo & Arco, 2021; Kafy et al., 2021; Wang et al., 2019; Yang et al., 2020). At the same time, it should be noted that the variability observed for the NDVI and PV indices cannot be attributed solely to LULC urban and coverage system variability. It also depends, to a lesser extent, on whether rainfall was abundant or scarce during those months and drainage or irrigation systems during the study period (Li et al., 2002). The higher the rainfall, the higher the NDVI and PV values, because the vegetation is leafier and greener, and the lower the rainfall, the lower the NDVI and PV values, because the vegetation is drier.

On some occasions and during the day, cities have lower temperatures than rural areas, whereas at night, the opposite is true. This is because of the amount of solar radiation received (which is particularly high in areas with sparse vegetation and bare soil) and the use of impermeable materials. However, due to the shade provided by trees and buildings, rural areas receive more solar radiation than urban areas during the day. Furthermore, green areas with vegetation in urban areas improve cooling rates. In other words, when urban areas are not exposed to solar radiation, impermeable surfaces do not heat up, high doses of heat are not released, and the ambient temperature does not change (Lemus-Canovas et al., 2020; Li & Meng, 2018; Sun et al., 2014; Yang et al., 2020). At night, urban areas are hotter than rural areas; because rural areas have little thermal inertia, they cool down quickly in the absence of solar radiation. As a result of the heat emitted by construction materials, urban areas remain warm, exacerbating the UHI phenomenon. As a result, the greater the density and population of a region, as well as the use of waterproof construction materials, the more heat these materials retain, and thus the more heat released into the atmosphere (Saaroni et al., 2018; Wu et al., 2019; Yang et al., 2020).

Temperatures in urban areas are higher in compact and industrial areas (LCZ-2, 3, and 10) than in open areas (LCZ-5 and 6). The configuration of LCZ-5 and 6 with buildings located at great distances and large green spaces with vegetation is once again motivating this circumstance. Studies have corroborated that vegetation has an altering effect on the temperature of urban areas, producing a cooling (Du et al., 2020; Qiu et al., 2017) that can range from 1 to 3°C. This effect is caused not only by shading and evapotranspiration processes, but also by the rates of cooling and heating caused by convection and transpiration. Our results are in line with those reported by other authors (Geletič et al., 2018; Martí Ezpeleta & Royé, 2021; Verdonck et al., 2018) who used the UrbClim climate model in their studies on other European cities.

The results show that relative humidity levels are higher in rural areas than in urban areas. The lowest humidity values detected here correspond to the compact and industrial zones (LCZ 2, 3, 10, and 6), which, as previously stated, have lower NDVI and PV indices and higher NDBI and UI values. These LCZs, however, have higher temperatures. The amount of vegetation within these LCZs and their evapotranspiration process, as well as the temperatures, could explain this situation. Higher temperature areas have lower relative humidity values (by definition, relative humidity is the ratio of absolute humidity to saturated humidity at room temperature; because the latter increases with temperature, a fixed value of absolute humidity produces lower humidity). This effect is corroborated by a study of four cities in Tennessee (USA), where trees were found to minimize the overall effect of heat due to an increase in environmental humidity (Hass et al., 2016). On the other hand, the study on the thermal comfort conditions on the historic squares of the cities of Seville and Madrid also confirms that the existence of high proportions of vegetation improves the thermal conditions of tourists who circulate through these public spaces (Karimi & Mohammad, 2022). Again, our results are in line with the findings of other authors (Geletič et al., 2018; Martí Ezpeleta & Royé, 2021; Verdonck et al., 2018) who applied the UrbClim climate model to study European cities.

The different LCZs in Seville show significant HI variability. Daytime heat stress can be classified as medium between 12:00 and 19:00 in all LCZs under normal environmental conditions. In contrast, the heat stress index can be classified as no risk at night and at dawn (10:00 p.m. and 9:00 a.m., respectively). It has been demonstrated that rural LZCs (LZC-B, D, and G) with more vegetation increase the HI faster in the morning (10:00 and 11:00), but decrease it faster in the afternoon than urban areas. During heat wave periods, there is a significant intensification of HI in the various LCZs, particularly between 1:00 p.m. and 7:00 p.m., when it is extremely hot. The HI intensifies rapidly in rural areas (LCZ-B, G, and D) in the mornings but decreases more rapidly than in urban areas in the afternoons. Taking this into account, the compact and industrial LCZs (LCZ-2, 3, and 10) have higher HI values and maintain the intensity for a longer period of time than the open zones (LCZ-5 and 6), which have a lower value in a shorter period of time. Because of the impermeable materials used in densely populated urban areas, accumulated heat is released into the atmosphere at night, maintaining high HI values and necessitating more time to mitigate its effects. This trend has been observed in the cities of Kolkata, Chennai, Delhi, Mumbai, and Nagpur (India), where the areas most built-up and having least vegetation cover correspond to the areas with the highest HI (Kotharkar et al., 2021; Kumar et al., 2022). Our results agree with previous reports (Geletič et al., 2018; Martí Ezpeleta & Royé, 2021; Verdonck et al., 2018). The regression model gave statistically significant and negative relationships between HI and the NDVI and PV indices, yet positive relationships with the NDBI, UI, and LULC variables. The results obtained in our research together with the similarities of other studies carried out in other cities through the use of the different LCZs (Geletič et al., 2018; Verdonck et al., 2018) may suggest that its use with the data from the UrbClim model may be adequate, not generating significant differences or a high contrast with the soil surface data used by the model and coming from the CORINE model.

Finally, UHS are more likely to be found in compact and industrial urban LCZs (LCZ 2, 3, and 10) than in rural and/or open LCZs (LCZ 5, 6, B, D, and G). It is reiterated that the LULC coverages with the highest proportion of UHS are built-up and farmland, while the coverages with the lowest percentages of UHS are water and vegetation. Researchers report that the areas with the highest temperatures include UHS areas due to a lack of green space and more impervious areas (Amindin et al., 2021; Guha et al., 2018; Hidalgo & Arco, 2021). Therefore, it is clearly essential to establish guidelines and policies that favor urban development in conjunction with a prevalence of green spaces and vegetation, or compact areas with roofs and plant facades that minimize the effects of temperatures. Heat wave periods are unfortunately bound to increase in frequency, intensity, and duration in the coming decades (Coumou et al., 2013; Hidalgo, 2021; Santamouris, 2020).

## Conclusions

The heat stress index of Seville (southern Spain) was evaluated in this study using data from the UrbClim climate model, which is part of the Copernicus Climate Change Service program and is implemented by the ECMWF. The well-known LCZ classification of ground surfaces was used to support the evaluation and allow extrapolation of the results to other urban areas. Our findings confirm that the majority of the population of Seville during the summer of 2017 lived in areas with a heat stress index classified as hot, then increasing to very hot under heat wave environmental conditions. Urban hot spots are defined as areas with the highest levels of heat stress. This environment had a number of negative effects on the population’s health and quality of life. In addition to confirming an important spatio-temporal variability between the HI and the LCZ—which increases significantly during episodes of intense heat—a positive correlation between the HI and the UI, LULC, and NDBI indices is identified, as well as a negative correlation with NDVI and PV. As a result, the HI is higher in compact and industrial LCZs (LCZ-2, 3, and 10) than in open LCZs and rural areas (LCZ-5, 6, D, B, and G), indicating greater resilience to heat waves. In general, areas with more impervious surfaces and fewer green spaces are more vulnerable to heat stress. Overall, these circumstances highlight the significance of designing future urban developments for open LZCs with large green spaces rather than closed LZCs. Public administrations and urban planners should work hard to improve cities’ resilience in the face of future extreme heat episodes. Contingency and urban climate control plans must be developed that encourage the use of green roofs and façades that reduce the rate of heat stress. Similarly, such measures will improve the quality of life for people who already live in densely populated areas. Our findings can be extrapolated to other cities or urban areas with similar LCZs. In terms of future research, new studies should extend the study period to evaluate the behavior of the heat stress index throughout the four seasons of the year, as well as analyze the evolution of this index over time. The severity of heat stress and its impact on population and urban growth in the coming decades must be assessed and predicted.

### Limitations to the study

This study has several limitations that need to be discussed: (1) It would be preferable to extend the heat wave study period to more years in order to confirm that the space-time evolution of the heat stress index maintains the evolution observed in 2017. (2) It is critical to conduct new studies on other cities in order to confirm that the results are similar to those reported here and, thus, guarantee the possibility of extrapolation to other cities through the use of LCZ. This situation acquires great importance considering that the surface data input of the UrbClim model comes from CORINE and our research has used the different LCZs. Therefore, it is necessary to evaluate the contrast between the coverage from CORINE and the different LCZs.

## Data Availability

Not applicable.

## References

[CR1] Alcock I, White MP, Lovell R, Higgins SL, Osborne NJ, Husk K, Wheeler BW (2015). What accounts for England’s green and pleasant land? A panel data analysis of mental health and land cover types in rural England. Landscape and Urban Planning.

[CR2] Amindin A, Pouyan S, Pourghasemi HR, Yousefi S, Tiefenbacher JP (2021). Spatial and temporal analysis of urban heat island using Landsat satellite images. Environmental Science and Pollution Research.

[CR3] An N, Dou J, González-Cruz JE, Bornstein RD, Miao S, Li L (2020). An observational case study of synergies between an intense heat wave and the urban heat island in Beijing. Journal of Applied Meteorology and Climatology.

[CR4] Anjos, M., Targino, A. C., Krecl, P., Oukawa, G. Y., & Braga, R. F. (2020). Analysis of the urban heat island under different synoptic patterns using local climate zones. *Building and Environment, 185*. 10.1016/j.buildenv.2020.107268

[CR5] Arbuthnott KG, Hajat S (2017). The health effects of hotter summers and heat waves in the population of the United Kingdom: A review of the evidence. Environmental Health: A Global Access Science Source.

[CR6] Arnfield AJ (2003). Two decades of urban climate research: A review of turbulence, exchanges of energy and water, and the urban heat island. International Journal of Climatology.

[CR7] Avdan, U., & Jovanovska, G. (2016). Algorithm for automated mapping of land surface temperature using LANDSAT 8 satellite data. *Journal of Sensors, 2016*. 10.1155/2016/1480307

[CR8] Brooke Anderson G, Bell ML, Peng RD (2013). Methods to calculate the heat index as an exposure metric in environmental health research. Environmental Health Perspectives.

[CR9] Brousse O, Georganos S, Demuzere M, Vanhuysse S, Wouters H, Wolff E, Linard C, van Lipzig NPM, Dujardin S (2019). Using local climate zones in Sub-Saharan Africa to tackle urban health issues. Urban Climate.

[CR10] Campbell J (1996). Introduction to remote sensing.

[CR11] Chen Y, Li X, Zheng Y, Guan Y, Liu X (2011). Estimating the relationship between urban forms and energy consumption: A case study in the Pearl River Delta, 2005-2008. Landscape and urban planning.

[CR12] Coumou D, Robinson A, Rahmstorf S (2013). Global increase in record-breaking monthly-mean temperatures. Climatic Change.

[CR13] De Ridder K, Lauwaet D, Maiheu B (2015). UrbClim - A fast urban boundary layer climate model. Urban Climate.

[CR14] Diallo-Dudek, J., Lacaze, B., & Comby, J. (2015). Land surface temperature in the urban area of Lyon metropolis: A comparative study of remote sensing data and MesoNH model simulation. 2015 Joint Urban Remote Sensing Event, JURSE 2015, 2–5. 10.1109/JURSE.2015.7120528

[CR15] Du J, Xiang X, Zhao B, Zhou H (2020). Impact of urban expansion on land surface temperature in Fuzhou, China using Landsat imagery. Sustainable Cities and Society.

[CR16] Dwivedi A, Mohan BK (2018). Impact of green roof on micro climate to reduce urban heat island. Remote Sensing Applications: Society and Environment.

[CR17] Emmanuel R, Krüger E (2012). Urban heat island and its impact on climate change resilience in a shrinking city: The case of Glasgow, UK. Building and Environment.

[CR18] Equere, V., Mirzaei, P. A., & Riffat, S. (2020). Definition of a new morphological parameter to improve prediction of urban heat island. *Sustainable Cities and Society, 56*. 10.1016/j.scs.2020.102021

[CR19] Fang L, Tian C (2020). Construction land quotas as a tool for managing urban expansion. Landscape and urban planning.

[CR20] Gago EJ, Roldan J, Pacheco-Torres R, Ordóñez J (2013). The city and urban heat islands: A review of strategies to mitigate adverse effects. Renewable and Sustainable Energy Reviews.

[CR21] Geletič J, Lehnert M, Savić S, Milošević D (2018). Modelled spatiotemporal variability of outdoor thermal comfort in local climate zones of the city of Brno, Czech Republic. Science of the Total Environment.

[CR22] Guha S, Govil H, Dey A, Gill N (2018). Analytical study of land surface temperature with NDVI and NDBI using Landsat 8 OLI and TIRS data in Florence and Naples city, Italy. European Journal of Remote Sensing.

[CR23] Hass, A. L., Ellis, K. N., Mason, L. R., Hathaway, J. M., & Howe, D. A. (2016). Heat and humidity in the city: Neighborhood heat index variability in a mid-sized city in the Southeastern United States. *International Journal of Environmental Research and Public Health, 13*(1). 10.3390/ijerph13010117 10.3390/ijerph13010117 PMC4730508 26761021

[CR24] Hidalgo D (2021). Analysis of synergies between the urban heat island and heat waves using Sentinel 3 satellite images: Study of Andalusian cities (Spain). Earth Systems and Environment.

[CR25] Hidalgo, D., & Arco, J. (2021). Modeling of the urban heat island on local climatic zones of a city using Sentinel 3 images: Urban determining factors. *Urban Climate, 37*. 10.1016/j.uclim.2021.100840

[CR26] Hidalgo-García, D., & Arco-Díaz, J. (2022). Modeling the surface urban heat island (SUHI) to study of its relationship with variations in the thermal field and with the indices of land use in the metropolitan area of Granada (Spain). *Sustainable Cities and Society, 87*. 10.1016/j.scs.2022.104166

[CR27] IPCC. (2021). The sixth report of the Intergovernmental Panel on Climate Change (IPCC). https://www.ipcc.ch/assessment-report/ar6/

[CR28] Jacobs C, Singh T, Gorti G, Iftikhar U, Saeed S, Syed A, Abbas F, Ahmad B, Bhadwal S, Siderius C (2019). Patterns of outdoor exposure to heat in three South Asian cities. Science of the Total Environment.

[CR29] Kafy A-A, Faisal AA, Rahman MS, Islam M, Al Rakib A, Islam MA, Khan MHH, Sikdar MS, Sarker MHS, Mawa J, Sattar GS (2021). Prediction of seasonal urban thermal field variance index using machine learning algorithms in Cumilla, Bangladesh. Sustainable Cities and Society.

[CR30] Karakuş CB (2019). The impact of land use/land cover (LULC) changes on land surface temperature in Sivas City Center and its surroundings and assessment of urban heat island. Asia-Pacific Journal of Atmospheric Sciences.

[CR31] Karimi A, Mohammad P (2022). Effect of outdoor thermal comfort condition on visit of tourists in historical urban plazas of Sevilla and Madrid. Environmental Science and Pollution Research.

[CR32] Kawamura M, Jayamana S, Tsujiko Y (1996). Relation between social and environmental conditions in Colombo Sri Lanka and the urban index estimated by satellite remote sensing data. International Archieve of Photogrammetry and Remote Sensing.

[CR33] Khamchiangta D, Dhakal S (2019). Physical and non-physical factors driving urban heat island: Case of Bangkok Metropolitan Administration, Thailand. Journal of Environmental Management.

[CR34] Kotharkar, R., Ghosh, A., & Kotharkar, V. (2021). Estimating summertime heat stress in a tropical Indian city using local climate zone (LCZ) framework. *Urban Climate, 36*. 10.1016/j.uclim.2021.100784

[CR35] Kovats RS, Campbell-Lendrum D, Matthies F (2005). Climate change and human health: Estimating avoidable deaths and disease. Risk Analysis.

[CR36] Kumar, P., Rai, A., Upadhyaya, A., & Chakraborty, A. (2022). Analysis of heat stress and heat wave in the four metropolitan cities of India in recent period. *Science of the Total Environment, 818*. 10.1016/j.scitotenv.2021.151788 10.1016/j.scitotenv.2021.151788 34826457

[CR37] Lau NC, Nath MJ (2012). A model study of heat waves over North America: Meteorological aspects and projections for the twenty-first century. Journal of Climate.

[CR38] Lemus-Canovas M, Martin-Vide J, Moreno-Garcia MC, Lopez-Bustins JA (2020). Estimating Barcelona’s metropolitan daytime hot and cold poles using Landsat-8 land surface temperature. Science of the Total Environment.

[CR39] Li B, Tao S, Dawson RW (2002). Relations between AVHRR NDVI and ecoclimatic parameters in China. International Journal of Remote Sensing.

[CR40] Li J, Song C, Cao L, Zhu F, Meng X, Wu J (2011). Impacts of landscape structure on surface urban heat islands: A case study of Shanghai, China. Remote Sensing of Environment.

[CR41] Li T, Meng Q (2018). A mixture emissivity analysis method for urban land surface temperature retrieval from Landsat 8 data. Landscape and Urban Planning.

[CR42] Martí Ezpeleta A, Royé D (2021). Intensidad y duración del estrés térmico en verano en el área urbana de Madrid. Geographicalia.

[CR43] Meehl GA, Tebaldi C (2004). More intense, more frequent, and longer lasting heat waves in the 21st century. Science.

[CR44] Mora C, Dousset B, Caldwell IR, Powell FE, Geronimo RC, Bielecki CR, Counsell CWW, Dietrich BS, Johnston ET, Louis LV, Lucas MP, Mckenzie MM, Shea AG, Tseng H, Giambelluca TW, Leon LR, Hawkins E, Trauernicht C (2017). Global risk of deadly heat. Nature Climate Change.

[CR45] Ngarambe J, Nganyiyimana J, Kim I, Santamouris M, Young Yun G (2020). Synergies between urban heat island and heat waves in Seoul: The role of wind speed and land use characteristics. PLoS One.

[CR46] Otukei, J. R., & Blaschke, T. (2010). Land cover change assessment using decision trees, support vector machines and maximum likelihood classification algorithms. *International Journal of Applied Earth Observation and Geoinformation,**12*(SUPPL. 1). 10.1016/j.jag.2009.11.002

[CR47] Qiu GY, Zou Z, Li X, Li H, Guo Q, Yan C, Tan S (2017). Experimental studies on the effects of green space and evapotranspiration on urban heat island in a subtropical megacity in China. Habitat International.

[CR48] Rajeshwari A (2014). Estimation of land surface temperature of Dindigul District using Landsat 8 data. International Journal of Research in Engineering and Technology.

[CR49] Rothfusz, L. P., & Headquarters, N. S. R. (1990). The heat index equation (or, more than you ever wanted to know about heat index). Fort Worth, Texas: National Oceanic and Atmospheric Administration, National Weather Service, Office of Meteorology, 23–90. papers://c6bd9143-3623-4d4f-963f-62942ed32f11/Paper/p395

[CR50] Royé, D., Sera, F., Tobías, A., Lowe, R., Gasparrini, A., Pascal, M., De’Donato, F., Nunes, B., & Teixeira, J. P. (2021). Effects of hot nights on mortality in Southern Europe. *Epidemiology*, 487–498. 10.1097/EDE.0000000000001359 10.1097/EDE.0000000000001359 33935136

[CR51] Saaroni H, Amorim JH, Hiemstra JA, Pearlmutter D (2018). Urban green infrastructure as a tool for urban heat mitigation: Survey of research methodologies and findings across different climatic regions. Urban Climate.

[CR52] Santamouris, M. (2020). Recent progress on urban overheating and heat island research. Integrated assessment of the energy, environmental, vulnerability and health impact. Synergies with the global climate change. *Energy and Buildings, 207*. 10.1016/j.enbuild.2019.109482

[CR53] Schneider A, Friedl MA, Potere D (2010). Mapping global urban areas using MODIS 500-m data: New methods and datasets based on “urban ecoregions”. Remote Sensing of Environment.

[CR54] Shafizadeh-Moghadam H, Weng Q, Liu H (2020). Modeling the spatial variation of urban land surface temperature in relation to environmental and anthropogenic factors: A case study of Tehran, Iran. GIScience and Remote sensing.

[CR55] Shafri HZ, Ramle FS (2009). 64-70.pdf. Information Technology Journal.

[CR56] Sharma R, Pradhan L, Kumari M, Bhattacharya P (2021). Assessing urban heat islands and thermal comfort in Noida City using geospatial technology. Urban Climate.

[CR57] Solecki WD, Rosenzweig C, Parshall L, Pope G, Clark M, Cox J, Wiencke M (2005). Mitigation of the heat island effect in urban New Jersey. Environmental Hazards.

[CR58] Song J, Chen W, Zhang J, Huang K, Hou B, Prishchepov AV (2020). Effects of building density on land surface temperature in China: Spatial patterns and determinants. Landscape and Urban Planning.

[CR59] Stewart, I., & Oke, T. (2009). Classifying urban climate field sites by “local climate zones”: The case of Nagano, Japan. The Seventh International Conference on Urban Climate, July, 1–5.

[CR60] Stewart ID, Oke TR (2012). Local climate zones for urban temperature studies. Bulletin of the American Meteorological Society.

[CR61] Sun Y, Zhang X, Zwiers FW, Song L, Wan H, Hu T, Yin H, Ren G (2014). Rapid increase in the risk of extreme summer heat in Eastern China. Nature Climate Change.

[CR62] UN. (2018). 68% of the world population projected to live in urban areas by 2050, says UN. https://www.un.org/development/desa/en/news/population/2018-revision-of-world-urbanization-prospects.html

[CR63] Verdonck ML, Demuzere M, Hooyberghs H, Beck C, Cyrys J, Schneider A, Dewulf R, Van Coillie F (2018). The potential of local climate zones maps as a heat stress assessment tool, supported by simulated air temperature data. Landscape and Urban Planning.

[CR64] Wang J, Ouyang W (2017). Attenuating the surface urban heat island within the local thermal zones through land surface modification. Journal of Environmental Management.

[CR65] Wang T, Shi J, Ma Y, Husi L, Comyn-Platt E, Ji D, Zhao T, Xiong C (2019). Recovering land surface temperature under cloudy skies considering the solar-cloud-satellite geometry: Application to MODIS and Landsat-8 data. Journal of Geophysical Research: Atmospheres.

[CR66] Wu, P., Yin, Z., Yang, H., Wu, Y., & Ma, X. (2019). Reconstructing geostationary satellite land surface temperature imagery based on a multiscale feature connected convolutional neural network. *Remote Sensing, 11*(3). 10.3390/rs11030300

[CR67] Yang, C., Wang, R., Zhang, S., Ji, C., & Fu, X. (2019). Characterizing the hourly variation of urban heat islands in a snowy climate city during summer. *International Journal of Environmental Research and Public Health, 16*(14). 10.3390/ijerph16142467 10.3390/ijerph16142467 PMC6678815 31373326

[CR68] Yang C, Yan F, Zhang S (2020). Comparison of land surface and air temperatures for quantifying summer and winter urban heat island in a snow climate city. Journal of Environmental Management.

[CR69] Yoo C, Han D, Im J, Bechtel B (2019). Comparison between convolutional neural networks and random forest for local climate zone classification in mega urban areas using Landsat images. ISPRS Journal of Photogrammetry and Remote Sensing.

[CR70] Yu X, Guo X, Wu Z (2014). Land surface temperature retrieval from Landsat 8 TIRS-comparison between radiative transfer equation-based method, split window algorithm and single channel method. Remote Sensing.

[CR71] Zha Y, Gao J, Ni S (2003). Use of normalized difference built-up index in automatically mapping urban areas from TM imagery. International Journal of Remote Sensing.

[CR72] Zhou D, Zhao S, Zhang L, Sun G, Liu Y (2015). The footprint of urban heat island effect in China. Scientific Reports.

